# Comparative effects of different exercise modalities on executive function in children with attention-deficit/hyperactivity disorder: a systematic review and network meta-analysis

**DOI:** 10.3389/fpsyg.2026.1786104

**Published:** 2026-04-30

**Authors:** Ling Ge, Yongsheng Sun

**Affiliations:** Capital University of Physical Education and Sports, Beijing, China

**Keywords:** ADHD, coordinative training, executive function, exercise interventions, inhibitory control, network meta-analysis

## Abstract

**Background:**

Exercise interventions have been increasingly identified as potential non-pharmacological approaches to ameliorate cognitive and behavioural performance among children with Attention-Deficit/Hyperactivity Disorder (ADHD). Nonetheless, the relative efficacy of various exercise modalities is still uncertain.

**Methods:**

This network meta-analysis and systematic review combined 19 trials (*N* = 845) to compare the impact of structured exercise on executive function and other outcomes. Four databases were searched: Scopus, PubMed, Web of Science, and SportDiscus. Randomised and controlled trials published between 2010 and 2025 were included. The main outcomes included working memory, inhibitory control, and cognitive flexibility; secondary outcomes included ADHD symptoms and motor performance. Random-effects models were used to pool standardised mean differences (SMDs). Only inhibitory control accuracy could be analysed with a Bayesian network meta-analysis (*k* = 14). *I*^2^, prediction intervals, design-by-treatment models, and GRADE were used to evaluate heterogeneity, inconsistency, and certainty of evidence.

**Findings:**

Effects were mixed across domains of executive functions. The overall effect of inhibitory control reaction time was not significant (SMD = −0.64, 95% CI: −1.72 to 0.44; *I*^2^ = 94%). The accuracy of working memory was also non-significant (SMD = 0.34, 95% CI: −0.26 to 0.94; *I*^2^ = 78%), but it was highly variable (prediction interval −1.22 to 1.90). There was little evidence of benefit in cognitive flexibility accuracy (SMD = 0.20, 95% CI: −1.51 to 1.91; *I*^2^ = 93%). The network meta-analysis found coordinative training to be the most effective modality for inhibitory control accuracy, performing better than the seven comparators (mean SMD ≈ 1.77). Direct comparisons indicated large, significant effects compared to waitlist (SMD = 2.21, 95% CI: 1.14–3.27) and attention control (SMD = −2.12, 95% CI: −3.51 to −0.72). There was a small, marginally significant improvement in ADHD symptoms (SMD = −0.43, 95% CI: −0.89 to 0.02; *I*^2^ = 6%). Moderation analyses revealed no significant dose–response or age effects, but moderate-dose programmes (481–2,880 min) demonstrated the most consistent benefits (SMD = 1.06, 95% CI: 0.32–1.79).

**Conclusion:**

Coordinative training produces the most consistent gains in inhibitory control among children with ADHD, and gains in more widely studied executive functions are inconclusive due to high variability and sparse networks. Modality-specific recommendations should be reinforced by more powerful head-to-head trials.

## 1 Introduction

Attention-deficit/hyperactivity disorder (ADHD) is a common disorder among children affecting about 5–7% of children globally (Tao et al., 2025). The disorder is characterised by persistent patterns of lack of concentration and attention, hyperactivity, and impulsivity that result in significant functional impairment of affected children in multiple domains. Individuals diagnosed with ADHD often exhibit deficits in executive function. Executive function is a domain of cognitive function that encompasses higher-order cognitive processes, including working memory, inhibitory control, and cognitive flexibility (Tao et al., 2025; Logan and Lim, 2025). Impairments in executive function are linked with academic, social, and behavioural difficulties among children diagnosed with ADHD (Tao et al., 2025; Hernández Ahumada et al., 2025; Zhu et al., 2023). Interventions targeting children with ADHD should focus beyond the management of core symptoms, but should also focus on improving executive function.

Executive function deficits are a core impairment among children with ADHD, though their severity and profile vary considerably across individuals (Tao et al., 2025; Logan and Lim, 2025). Working memory is a component of executive function and is defined as the ability to temporarily hold and manipulate information (Al-Saad et al., 2021; Chai et al., 2018). Children with ADHD are frequently shown to have deficits in working memory, attributed to difficulties with handling difficult tasks that require coherent concentration and mental manipulation of information (Al-Saad et al., 2021; Chai et al., 2018). Inhibitory control, on the other hand, is denoted by the ability to suppress inclination towards distractions and/or inappropriate responses. Inhibitory control is deficient, manifested through impulsive behaviour and difficulty maintaining attention in the presence of competing stimuli (Chai et al., 2018; Kostyrka-Allchorne et al., 2023; Kofler et al., 2024). Cognitive flexibility is another domain of executive function that is impaired among children with ADHD (Revollo Carrillo et al., 2025; Lei et al., 2022). Cognitive flexibility is the ability to shift between mental sets as well as adapt to tasks of varying concentration and mental capacity needs. Impaired cognitive flexibility, as expressed among children with ADHD, manifests as perseverative behaviour and difficulty transitioning between activities (Chai et al., 2018; Revollo Carrillo et al., 2025; Lei et al., 2022). The heterogeneity in executive function profiles across individuals with ADHD underscores the importance of interventions that can address these diverse cognitive needs.

Evidence-based treatment and management of ADHD has primarily focused on pharmacological interventions; however, there is a growing interest and demand for alternative non-pharmacological interventions, with a keen interest in physical activity and exercise interventions. Several systematic reviews and meta-analyses have shown promising evidence for physical activity and exercise interventions in the management of ADHD symptoms and their associated deficits in executive function (Zhu et al., 2023; Kostyrka-Allchorne et al., 2023; Verburgh et al., 2014). Exercise interventions have been proposed to improve executive functioning in individuals with ADHD through multiple neurobiological mechanisms. These mechanisms include, but are not limited to, increased cerebral blood flow, upregulation of neurotrophic factors such as brain-derived neurotrophic factor, enhanced neurogenesis and angiogenesis, and modulation of neurotransmitter systems implicated in attention and cognitive control (Hernández Ahumada et al., 2025; Kostyrka-Allchorne et al., 2023; Revollo Carrillo et al., 2025; Verburgh et al., 2014). These mechanisms indicate the ability of physical exercise to modulate neural systems affecting executive function, hence exhibiting potential benefits in enhancing and managing executive function capacity among children with ADHD.

Recent systematic reviews and meta-analyses have reported that exercise interventions can positively contribute to executive function enhancement in children with ADHD, with effect sizes ranging from moderate to large across different executive function domains (Zhu et al., 2023; Lei et al., 2022; Verburgh et al., 2014; Song et al., 2025; Tao et al., 2025; Wang et al., 2024; Contreras-Osorio et al., 2021). However, the literature is predominantly focused on comparing exercise with non-exercise interventions as opposed to head-to-head comparisons between exercise and exercise interventions. This creates a critical gap in our understanding of which specific exercise modalities may be most effective for improving executive function domains.

This heterogeneity in exercise interventions research poses significant challenges to clinicians, educators, and families who seek to make informed decisions about the optimal physical activity programme for their children with ADHD (Hernández Ahumada et al., 2025; Al-Saad et al., 2021; Contreras-Osorio et al., 2021). Various exercise modalities have diverse effects on neurobiological systems and engage different executive functioning processes, resulting in varying effects on ADHD management. For example, open-skill exercises are dynamic and demand intimate involvement and concentration, which may place greater demands on inhibitory control and cognitive flexibility compared to closed exercises performed in a stable and predictable environment (Zhu et al., 2023). In addition, mind–body interventions that focus on breath regulation and mindfulness can benefit attentional regulation and cognitive integrative tasks that combine both physical and cognitive processes, hence yielding different effects on executive control (Song et al., 2025; Tao et al., 2025). These differential effects are critical in understanding optimal physical exercise intervention recommendations for executive function.

Network meta-analysis offers a strong analytical paradigm to answer this question by integrating both direct and indirect evidence to approximate the relative efficacy of two or more interventions at the same time (Freeman et al., 2019; Hutton et al., 2015). This method allows prioritising of various exercise modalities and offers strong evidence to inform clinical practice guidelines and school-based interventions. Network meta-analysis can be used to determine which types of exercises are most effective in producing particular outcomes in executive functions, even when direct head-to-head comparisons in the literature are scarce, by combining all available evidence about different exercise interventions (Verburgh et al., 2014; Tao et al., 2025; Wang et al., 2024; Hutton et al., 2015; Veroniki et al., 2021). Such an approach is especially useful due to the practical limitations that prevent the implementation of large-scale trials directly comparing all possible exercise modalities.

Moreover, knowledge of whether treatment effects depend on characteristics of interventions, including supervision level, session length, frequency of intervention, and length of programme, will be essential information to inform the best exercise interventions in children with ADHD. Dose–response relationships in exercise interventions are still only poorly understood, and recent evidence indicates that cognitive benefits can be non-linear and dependent on exercise intensity and cognitive domain studied (Logan and Lim, 2025; Lei et al., 2022; Tao et al., 2025). Also, individual traits, such as age, sex, ADHD subtype, baseline severity, and comorbidities, can moderate intervention effects, which implies that exercise prescription should be individualised. Existing clinical practice guidelines include little specific guidance on exercise interventions despite increasing evidence of their effectiveness, which is a translational gap that this review will fill.

Therefore, the present systematic review and network meta-analysis addresses this evidence gap by comparing the efficacy of aerobic exercise training, cognitively engaging training, coordinative training, mind–body training, combined or mixed training, and resistance training on executive function outcomes in children and adolescents with ADHD. The three primary outcomes of interest are working memory, inhibitory control, and cognitive flexibility. Secondary outcomes include ADHD symptom severity, motor performance, and treatment acceptability. By synthesising evidence from randomised controlled trials and controlled clinical trials, this review aims to provide evidence-based guidance on exercise prescription for children with ADHD and to inform future research priorities in this rapidly growing field.

## 2 Methods

### 2.1 Protocol registration and reporting standards

This systematic review and network meta-analysis protocol was registered with the International Platform of Registered Systematic Review and Meta-Analysis Protocols (INPLASY) on 12 January 2026 (Registration No.: INPLASY202610036; DOI: 10.37766/inplasy2026.1.0036).^1^ It is acknowledged that protocol registration was completed retrospectively following completion of the review; this was due to administrative requirements at the time of submission and represents a limitation with respect to prospective registration standards. This review is reported in accordance with the Preferred Reporting Items for Systematic Reviews and Meta-Analyses extension for network meta-analyses (PRISMA-NMA) (Hutton et al., 2015; Veroniki et al., 2021) and the PRISMA 2020 statement (Page et al., 2021).

As part of the review process, two amendments to the registered protocol were submitted. First, the search was conducted across four electronic databases, namely, PubMed, Scopus, Web of Science Core Collection, and SPORTDiscus, as they provided sufficient coverage of the relevant literature. Second, network meta-analysis was feasible only for inhibitory control accuracy due to insufficient network connectivity in other outcome domains; remaining outcomes were analysed using conventional pairwise meta-analysis. These deviations are acknowledged as limitations of the review.

### 2.2 Eligibility criteria

The predefined criteria used to evaluate study eligibility were founded on the Population, Intervention, Comparison, Outcome, and Study design (PICOS) framework (Amir-Behghadami and Janati, 2020; Higgins et al., 2024). Participants were children and adolescents aged 6–18 years with clinically diagnosed ADHD by standardised diagnostic criteria (Augustin et al., 2024; Cianchetti, 2020). Interventions were structured exercise training programmes, performed in series, and were classified as aerobic exercise, resistance training, coordinative activities, mind–body exercises, cognitively engaging physical activities, or multimodal programmes (Zhu et al., 2023; Song et al., 2025; Tao et al., 2025; Wang et al., 2024). Comparisons were made with usual care, wait-list control, attention control, or other exercise modalities. The interested outcomes were executive function domains (working memory, inhibitory control, cognitive flexibility), ADHD symptoms, and physical fitness, assessed using validated measures (Logan and Lim, 2025; Hernández Ahumada et al., 2025; Verburgh et al., 2014). Only randomised controlled trials and controlled clinical trials with concurrent control groups were included as study designs. There are specific inclusion and exclusion criteria as follows.

#### 2.2.1 Inclusion criteria

The inclusion criteria were as follows: randomised controlled trials or controlled clinical trials; parallel-group or crossover design; child and adolescent participants aged 6–18 years; clinical diagnosis of ADHD using standardised criteria (DSM-IV, DSM-5, ICD-10, ICD-11); structured exercise interventions administered on multiple occasions; measurement of at least one domain of executive functioning using validated neuropsychological tests or standardised rating scales; peer-reviewed journal articles; English language publications; publication dates between January 2010 and 2025.

#### 2.2.2 Exclusion criteria

Studies were filtered out when they satisfied any of the following criteria: non-randomised study designs: observational, case series, and single-group pre-post studies; participants aged 18 years and above; primary diagnosis of intellectual disability, autism spectrum disorder, or other neurodevelopmental conditions; single-session acute exercise interventions or unstructured physical activity recommendations; intervention programmes lacking explicit protocols in terms of frequency, duration, and intensity; outcomes only on ADHD symptoms without assessing executive functions; conference abstracts with inadequate methodological description; and inadequate statistical data to include.

### 2.3 Database search strategy

A systematic search was conducted in four electronic databases: PubMed, Scopus, Web of Science Core Collection, and SPORTDiscus (Bramer et al., 2017). The formal search was executed on 1 December 2025, covering publications from January 2010 to 1 December 2025. These databases were selected to provide complementary coverage of the biomedical, interdisciplinary, and exercise science literature relevant to paediatric ADHD and exercise intervention research. Search strategies were developed using the PICO framework, which included four concept areas (Bramer et al., 2017; DeMars and Perruso, 2022; Leblanc et al., 2024): (1) ADHD populations (e.g., attention deficit hyperactivity disorder, ADHD, hyperkinetic disorder), (2) paediatric age groups (e.g., child, adolescent, youth, paediatric), (3) exercise interventions (e.g., exercise, physical activity, aerobic, resistance training, yoga, coordinative training), and (4) executive function outcomes (e.g., executive function, working memory, inhibitory control, cognitive flexibility). The search used controlled vocabulary (MeSH terms in MEDLINE) and free-text terms in title, abstract, and keywords using Boolean operators (AND, OR). Randomised controlled trials and controlled clinical trials were filtered by study design to increase precision. MEDLINE search strategy was pilot-tested, refined, and adjusted to the syntax and indexing structure of each database without conceptual compromise. Full search terms with database-specific variations are provided in Appendix 1.

### 2.4 Study screening and selection

A two-stage screening process was conducted to select the studies used in the analysis. All citations from the searched databases were imported into Zotero to be managed and initially organised (Tsou et al., 2020; Waffenschmidt et al., 2023). The structured references were then exported to EPPI-Reviewer to undergo automated duplicate screening and removal. Automated duplicate removal was complemented with manual verification to detect near-duplicate entries (Tsou et al., 2020; Mahtani et al., 2020; Polanin et al., 2019). Systematic screening of deduplicated references was then performed. All records were screened by two reviewers in two stages: (1) title and abstract screening against predefined eligibility criteria, followed by (2) full-text review of potentially eligible studies (Page et al., 2021; Higgins et al., 2024; Rožanc and Mernik, 2021). Reviewers used hierarchical coding systems with mutually exclusive categories at each stage, assigning one code per article when the first relevant inclusion or exclusion criterion was located (Tsou et al., 2020; Mahtani et al., 2020; Polanin et al., 2019). Differences between reviewers were addressed by discussing to agree. A senior reviewer offered final arbitration when consensus was not possible. The PRISMA flow diagram was facilitated by EPPI-Reviewer to support independent screening processes, automated conflict detection, and systematic documentation of exclusion reasons with frequencies.

### 2.5 Data extraction

Two reviewers independently extracted data using standardised forms constructed specifically to be used in this review and piloted on a subset of included studies. The extraction framework included six interconnected tables that systematically described study design features, participant demographic and clinical features, specific intervention parameters, primary executive function results, secondary results, and risk of bias evaluations (Rožanc and Mernik, 2021; Büchter et al., 2020; Mathes et al., 2017). Data extracted contained study identifiers, randomisation strategies, sample characteristics, ADHD diagnostic criteria, comorbidities, medication status, intervention type (exercise modality), dose parameters (frequency, duration, session length, intensity), supervision levels, setting characteristics, adherence rates, and detailed outcome data with means, standard deviations, sample sizes, and effect estimates.

In the case of the multiple executive function outcomes, each measure was assigned to separate rows in relation to each comparison, and the outcome domain (working memory, inhibitory control, cognitive flexibility), measurement tool, rater types (performance-based scales versus rating scales), and scoring directions were carefully considered. Multi-arm trials were denoted with letter suffixes (a, b, c) to distinguish comparisons but to record shared control groups to facilitate the handling of meta-analyses (Büchter et al., 2020; Rücker et al., 2017). Established formulae were used to convert standard error and confidence interval to standard deviations, and all calculations were recorded.

### 2.6 Outcome measures and assessment

#### 2.6.1 Principal executive function outcomes

The main results included executive function scores measured by validated neuropsychological tests or standardised behavioural rating scales. The domains of interest in executive functions were working memory, measured by tasks like Digit Span, Spatial Span, or n-back paradigms; inhibitory control, measured by the Stroop Test, Go/No-Go tasks, or Stop Signal Task; and cognitive flexibility, measured by the Wisconsin Card Sorting Test, Trail Making Test, or task-switching paradigms (Al-Saad et al., 2021; Chai et al., 2018; Kostyrka-Allchorne et al., 2023; Kofler et al., 2024; Lei et al., 2022). Both performance-based neuropsychological and behavioural rating scales, including the Behaviour Rating Inventory of Executive Function (BRIEF) and parent-report, teacher-report, and self-report versions, were incorporated as the primary outcomes. Outcome assessment timing was limited to immediate post-intervention or within 4 weeks of intervention completion to measure the proximal effects of exercise training on executive function. To be included in quantitative synthesis, studies had to provide adequate statistical data, such as means, standard deviations, and sample sizes, or effect sizes with confidence intervals for at least one executive function primary outcome.

Where studies reported both performance-based neuropsychological measures and behavioural rating scales for the same executive function domain, these were analysed separately and not combined within a single meta-analytic estimate, given that they measure related but conceptually distinct constructs, direct cognitive performance versus informant-perceived behaviour. Pooled estimates in this review reflect performance-based outcomes. The inclusion of rating scales alongside performance measures in the eligibility criteria was retained to maximise inclusivity, but sensitivity to measurement type is acknowledged in the interpretation of results.

Where a study reported multiple performance measures within the same executive function domain (e.g., both Stroop colour-word and Go/No-Go accuracy contributing to inhibitory control accuracy), the outcome most central to the domain construct, as determined by the primary outcome designation in the original study or, where unspecified, by *a priori* consensus between reviewers, was selected for inclusion. This rule was applied to avoid unit-of-analysis errors arising from multiple correlated outcomes from a single sample contributing to the same pooled estimate (Büchter et al., 2020; Rücker et al., 2017).

#### 2.6.2 Secondary outcomes

Additional outcomes were ADHD symptoms severity measured with standardised rating scales, including the ADHD Rating Scale, Conners Rating Scales, or Vanderbilt Assessment Scales, distinguishing between parent-rated, teacher-rated, and self-reported symptoms; academic performance measures like standardised achievement test scores or teacher ratings of academic functioning; behavioural measures like on-task behaviour, classroom behaviour ratings, or social skills scales; physical fitness measures such as cardiovascular fitness assessed using graded exercise testing, muscular strength, or motor coordination skills; and mental health measures such as anxiety and depression symptoms Performance outcomes of motor performance measured with tools like the Movement Assessment Battery of Children (M-ABC) were also obtained (Al-Saad et al., 2021; Chai et al., 2018; Kostyrka-Allchorne et al., 2023; Kofler et al., 2024; Lei et al., 2022). These supplementary outcomes offered valuable background to the interpretation of executive function results and to the overall effects of exercise interventions on functioning in children with ADHD.

### 2.7 Risk of bias assessment

Two reviewers independently assessed the methodological quality of included studies using the revised Cochrane Risk of Bias tool for randomised trials (RoB 2) (Sterne et al., 2019). RoB 2 was applied to all 18 randomised controlled trials across five domains: bias arising from the randomisation process; bias due to deviations from intended interventions; bias due to missing outcome data; bias in outcome measurement; and bias in selection of reported results (Sterne et al., 2019; Moseley et al., 2019; Higgins et al., 2011). One study employed a non-randomised controlled design (Chang et al., 2014); RoB 2 was applied to this study for consistency of reporting across the review, with the caveat that its domain judgements, particularly for the randomisation process domain, were evaluated conservatively given the absence of random allocation. This study received an overall high risk of bias rating, the only study to do so, and was treated accordingly in sensitivity analyses and evidence certainty assessments. The more appropriate tool for non-randomised studies (ROBINS-I) was not applied, given the singular nature of this study in the sample; its influence on pooled estimates is addressed in the limitations. Reviewer disagreements were settled by discussion or third-reviewer arbitration.

### 2.8 Network meta-analysis

Outcome domains with adequate data (*n* ≥ 10 studies) were then subjected to network meta-analysis, and those with 5–9 studies to conventional pairwise meta-analysis. Standardised mean differences were the main effect measure of continuous executive function results, which were computed with post-intervention means, standard deviations, and sample sizes (Hutton et al., 2015; Veroniki et al., 2021; Higgins et al., 2024; Chaimani et al., 2024). Random-effects size models were used to explain anticipated heterogeneity across studies due to differences in characteristics of participants, intervention regimens, and measurement tools (Dettori et al., 2022; Röver et al., 2015; Seide et al., 2019).

Before conducting network meta-analysis, the transitivity assumption was evaluated, that is, whether the distribution of potential effect modifiers was sufficiently balanced across studies sharing a common comparator to justify indirect comparisons (Chaimani et al., 2024). Key modifiers considered included participant age range, ADHD diagnostic criteria, medication status, control condition type, and outcome measurement tool. Across the 14 studies contributing to the inhibitory control accuracy network, populations were consistently children aged 6–12 years with clinically diagnosed ADHD, control conditions were predominantly waitlist or attention controls, and outcomes were performance-based neuropsychological tests. Minor heterogeneity in medication status and outcome instruments was noted but judged insufficient to violate transitivity. The transitivity assumption is therefore considered plausible, though it cannot be formally tested in the absence of closed loops in the network, which precluded node-splitting analysis and net heat plot interpretation. This represents an inherent limitation of the network structure and is reflected in the certainty of evidence ratings.

Bayesian random-effect method was used to conduct network meta-analysis with MetaInsight (version 4.0) and netmeta and metafor packages in R (version 4.3.1) (Jiang et al., 2025; Nie et al., 2025; Weber et al., 2021). The Bayesian method employed vague prior distributions, where 50,000 iterations were used after 20,000 burn-in iterations to guarantee convergence. Network geometry was analysed using network plots illustrating all treatment comparisons and their relationships. Surface Under the Cumulative Ranking Curve (SUCRA) values in the Bayesian framework and P-scores in the frequentist approach were used to quantify treatment rankings, with a range of 0% (worse) to 100% (best) (Daly et al., 2019; Mbuagbaw et al., 2017; Laloux et al., 2025).

Statistical heterogeneity was evaluated using *I*^2^ statistics and tau^2^ (von Hippel, 2015; Zhao, 2013; Turner et al., 2015). To determine the possible sources of inconsistency within the network, network inconsistency was assessed using design-by-treatment interaction models and net heat plots (Freeman et al., 2019). When closed loops were present, node-splitting analysis was conducted to compare direct and indirect estimates of evidence. Comparison-adjusted funnel plots were used to evaluate publication bias when enough studies existed. Network findings were supplemented by pairwise meta-analyses to provide comparisons with sufficient direct evidence. Sensitivity analyses investigated how risk of bias ratings, intervention duration categories, and levels of supervision affected effect estimates.

Network meta-analysis was feasible only for inhibitory control accuracy [IC-Accuracy; *k* = 14 studies (Memarmoghaddam et al., 2016; Ludyga et al., 2023; Huang et al., 2025; Chang et al., 2014; Chang et al., 2022; Kadri et al., 2019; Liang et al., 2022; Liu et al., 2025; Nejati, 2021; Nejati and Derakhshan, 2021; Pan et al., 2016; Smith et al., 2019; Zhao et al., 2024)]. Other primary outcomes had insufficient studies for robust network estimation: inhibitory control reaction time (IC-RT) [*k* = 9, but sparse connectivity (Memarmoghaddam et al., 2016; Ludyga et al., 2023; Kadri et al., 2019; Liang et al., 2022; Liu et al., 2025; Smith et al., 2019; Benzing and Schmidt, 2019; Bustamante et al., 2016; Durgut et al., 2020)], working memory accuracy [*k* = 8 (Huang et al., 2025; Liang et al., 2022; Nejati, 2021; Nejati and Derakhshan, 2021; Benzing and Schmidt, 2019; Bustamante et al., 2016; Ludyga et al., 2022; Huang et al., 2024)], and cognitive flexibility accuracy [*k* = 5 (Chang et al., 2022; Liang et al., 2022; Nejati, 2021; Nejati and Derakhshan, 2021)]. Secondary outcomes, including ADHD symptoms [*k* = 5 (Huang et al., 2025; Nejati, 2021; Nejati and Derakhshan, 2021; Zhao et al., 2024; Bustamante et al., 2016)] and motor performance [*k* = 5 (Ludyga et al., 2023; Huang et al., 2025; Chang et al., 2014; Pan et al., 2016; Ludyga et al., 2022)], lacked adequate direct comparisons to form connected networks. The minimum threshold of 10 studies with sufficient network connectivity was met only by IC-Accuracy (Chaimani et al., 2024; Turner et al., 2015). This decision reflects the actual evidence base rather than a change in analytical intent, and the limited network connectivity for most domains is acknowledged as a fundamental constraint of the current literature.

### 2.9 Subgroup analysis

When enough studies were found, pre-specified subgroup analyses were performed in the Cochrane Review Manager (RevMan version 5.4) to investigate potential effect modifiers (Nair and Borkar, 2024). Parameters of intervention dosage were classified as: duration (short: <8 weeks, moderate: 8–12 weeks, long: >12 weeks), frequency (low: 1–2 sessions/week, moderate: 3–4 sessions/week, high: ≥5 sessions/week), and session length (short: <30 min, moderate: 30–60 min, long: >60 min) where total exercise dosage was calculated by multiplying duration, frequency and session (short = <480; moderate: 481–2,880; high = 2,881+). Age of participants was stratified into children (6–12 years) and adolescents (13–18 years) to consider the developmental differences in executive functioning maturation and exercise capacity. Chi-squared tests were used to assess subgroup differences with a statistical significance level of *p* < 0.10, recognising the low statistical power to detect subgroup effects. Subgroup analyses were performed only on outcome domains with sufficient study numbers and were interpreted with caution due to the nature of the observational comparisons and possible confounding by co-varying intervention characteristics.

### 2.10 Publication bias assessment

Comparison-adjusted funnel plots tailored to network meta-analysis were used to evaluate publication bias in outcome domains with at least 10 studies. The funnel plot asymmetry was visually inspected and complemented with the Egger regression test to test the small-study effects statistically (Afonso et al., 2024; Mathur and VanderWeele, 2020; Song et al., 2002). The comparison-adjusted methodology took into consideration the multi-arm nature of the network, plotting effect sizes versus their standard errors but conditioning on various comparisons within the network. Asymmetry in funnel plots, especially the lack of small studies with null or negative effects, indicated possible publication bias. Sensitivity analyses investigated how omitting small studies or high-risk-of-bias studies affected network estimates.

### 2.11 Certainty of evidence

Each outcome and treatment comparison underwent evaluation of evidence certainty using the Grading of Recommendations Assessment, Development and Evaluation (GRADE) methodology modified to network meta-analysis, which is implemented in GRADEPro GDT (version 3.6.1). The GRADE framework evaluated five areas: study limitations (risk of bias), inconsistency (heterogeneity and incoherence between direct and indirect evidence), indirectness (generalisability of evidence), imprecision (confidence interval width), and publication bias (Al Duhailib et al., 2024; Brozek et al., 2021; Cochrane Training, 2023). Certainty of evidence was classified as high, moderate, low, or very low, with high certainty assigned to randomised controlled trials and downgrades based on domain-specific issues. Network meta-analysis estimates included direct and indirect contributions to evidence, with certainty ratings representing the lower of the two pathways. Findings tables summarised effect estimates with their respective certainty ratings on key treatment comparisons and outcomes, enabling clear interpretation of the quality of evidence that supports clinical recommendations.

## 3 Results

### 3.1 Study screening and selection

**Figure 1:**
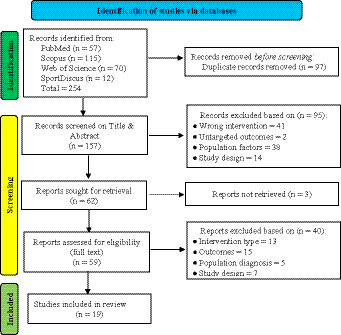
PRISMA flow diagram.

The systematic database search identified 254 records across four electronic databases (Figure 1). Following duplicate removal, 157 unique records underwent title and abstract screening, resulting in 62 reports proceeding to full-text assessment. Of these, 40 reports were excluded primarily due to inappropriate intervention types (*n* = 13), lack of executive function outcomes (*n* = 15), and unsuitable population characteristics (*n* = 5). Three reports could not be retrieved despite author contact attempts. The final review included 19 studies meeting all eligibility criteria for data extraction and synthesis (Memarmoghaddam et al., 2016; Ludyga et al., 2023; Huang et al., 2025; Chang et al., 2014; Chang et al., 2022; Kadri et al., 2019; Liang et al., 2022; Liu et al., 2025; Nejati, 2021; Nejati and Derakhshan, 2021; Pan et al., 2016; Smith et al., 2019; Zhao et al., 2024; Benzing and Schmidt, 2019; Bustamante et al., 2016; Durgut et al., 2020; Ludyga et al., 2022; Huang et al., 2024; Hattabi et al., 2022). The predominant exclusion reasons reflected the specificity of inclusion criteria, particularly the requirement for validated executive function assessment and structured exercise interventions in clinically diagnosed ADHD populations.

### 3.2 Study and population characteristics

The 19 eligible studies consisted of 18 randomised controlled trials (Memarmoghaddam et al., 2016; Ludyga et al., 2023; Huang et al., 2025; Chang et al., 2022; Kadri et al., 2019; Liang et al., 2022; Liu et al., 2025; Nejati, 2021; Nejati and Derakhshan, 2021; Pan et al., 2016; Smith et al., 2019; Zhao et al., 2024; Benzing and Schmidt, 2019; Bustamante et al., 2016; Durgut et al., 2020; Ludyga et al., 2022; Huang et al., 2024; Hattabi et al., 2022) and one controlled trial (Chang et al., 2014), which collectively enrolled 845 participants with ADHD (Table 1). Most studies originated from Asia [*n* = 8; China (Liang et al., 2022; Zhao et al., 2024; Huang et al., 2024), Taiwan (Huang et al., 2025; Chang et al., 2014; Chang et al., 2022; Pan et al., 2016), Hong Kong (Liu et al., 2025)], Europe [*n* = 4; Switzerland/Germany (Ludyga et al., 2023; Ludyga et al., 2022), *n* = 1; Switzerland (Benzing and Schmidt, 2019), *n* = 1; Turkey (Durgut et al., 2020)], Middle East [3 = Iran (Memarmoghaddam et al., 2016; Nejati, 2021; Nejati and Derakhshan, 2021)], and North America (*n* = 2; USA), and Africa [*n* = 2; Tunisia (Kadri et al., 2019; Hattabi et al., 2022)]. Sixteen studies were two-arm designs that compared a single exercise intervention to control conditions, and three studies were three-arm designs that could compare multiple interventions (Table 1). The study population was predominantly male (81.89%), which is indicative of traditional sex distributions in ADHD. The mean age of the participants was between 7.06 and 15.82 years, with 17 (89.5%) studies including children aged 6–12 years and two (10.5%) studies involving adolescents aged 13–18 years (Table 1).

**Table 1:** Study and population characteristics from the included studies.

Study ID	Age range	Mean age (SD)	Sample size	ADHD diagnostic criteria	Comorbidities allowed	Medication status	Medication during study
Benzing and Schmidt (2019)	8–12	CET: 10.46 (1.30); WLC: 10.39 (1.44)	CET: F = 4, M = 24; WLC: F = 4, M = 19	ICD-10	Excluded: neurological, Tourette, epilepsy	INT: 71.4%; CON: 73.9%	Continued
Bustamante et al. (2016)	6–12	AET: 9.4 (2.2); ACC: 8.7 (2.0)	AET: F = 6, M = 13; ACC: F = 5, M = 11	DSM-IV (DISC-IV interview)	ODD, CD allowed; 58% overweight/obese	INT: 7%; CON: 21% ever taken	1 child on MPH
Chang et al. (2014)	5–10	COMB: 8.19 (NR); WLC: 8.78 (NR)	COMB: F = 4, M = 10; CET: F = 3, M = 13; WLC: F = 0, M = 13	DSM-IV-TR	Excluded: neurological disorders	INT: 50%; CON: 46% on MPH	Withdrawn 24 h before testing
Chang et al. (2022)	Grade 1–6	COT: 8.31 (1.30); CET: 8.38 (1.20) CON: 8.38 (1.31)	COT: F = 3, M = 13; CON: F = 3, M = 13	DSM-5	Handwriting difficulties required	INT: 4 on MPH; CON: 4 on MPH	Continued
Durgut et al. (2020)	7–11	COM: 8.33 (1.34); AET: 8.27 (1.22)	COM: F = 3, M = 12; AET: F = 3, M = 12	DSM-5	None	Unmedicated	NR
Hattabi et al. (2022)	8–12	AET: 9.85 (1.53); WLT: 9.60 (1.79)	AET: F = 3, M = 17; WLT: F = 2, M = 20	DSM-5	None	Unmedicated	NR
Huang et al. (2024)	8–10	COT: 10.18 (1.10); WLT: 8.94 (0.56)	COT: M = 22; CON: M = 17	DSM-5	None	Medication-free ≥4 weeks	NR
Huang et al. (2025)	6–12	COT: 10.21 (1.27); WLT: 10.21 (1.24)	COT: M = 12; CON: M = 12	DSM-IV	None	17% on stable medication	NR
Kadri et al. (2019)	11–18	MBT: 14.5 (3.5); CON: 14.2 (3.0)	MBT: F = 2, M = 18; CON: F = 2, M = 18	DSM-IV/DSM-5	NR	NR	NR
Liang et al. (2022)	6–12	COMB: 8.37 ± 1.42; WLT: 8.29 ± 1.27	COMB: F = 10, M = 30; WLT: F = 8, M = 32	DSM-5; K-SADS-PL	None (excluded combined psychiatric disorders)	0% medicated	Medication-naive required (>6 months)
Liu et al. (2025)	12–17	AET: 15.82 (1.11); WLT: 13.65 (1.21)	AET: F = 5, M = 35; WLT: F = 3, M = 37	Clinical diagnosis (parent-reported)	None specified	0% medicated	Medication-naive required
Ludyga et al. (2022)	8–12	MBT: 10.0 ± 1.2; WLT: 10.8 ± 1.2	MBT: M = 23, F = 6; WLT: M = 18, F = 10	DSM-5	Excluded: ASD, structural epilepsy	Stable MPH/DEX ≥ 3 months required	Continued (MPH/DEX: MBT 26.7 ± 13.6 mg/d, WLT 30.3 ± 12.2 mg/d)
Ludyga et al. (2023)	8–12	MBT: 10.0 ± 1.2; WLT: 10.8 ± 1.2	MBT: M = 23, F = 6; WLT: M = 17, F = 10	DSM-5	Excluded: ASD, structural epilepsy	Stable MPH/DEX ≥ 3 months, no change in last month	Continued (28.5 ± 12.9 mg/day)
Memarmoghaddam et al. (2016)	7–11	COMB: 8.31 ± 1.29; CON: 8.29 ± 1.31	COMB: M = 19; M = 17	DSM-IV	Excluded: IQ < 70, autism, other mental disorders/health problems	Medication-naive (drug-free)	None
Nejati (2021)	7–11	CET: NR; AET: NR (combined 9.43 ± 1.43)	M = 26, F = 4	DSM-V	Excluded: head injury, seizure, other medical diagnoses	NR	NR
Pan et al. (2016)	6–12	COT: 8.93 ± 1.49; WLT: 8.87 ± 1.56	M = 32 (100%)	DSM-IV	Excluded: psychosis, PDD, brain injury	56% medicated	Maintained current treatment
Nejati and Derakhshan (2021)	7–12	CET: 9.73 ± 1.94; ACC: 9.21 ± 1.25	CET: M = 10, F = 5; ACC: M = 6, F = 8	Psychiatrist diagnosis	NR	NR	NR
Smith et al. (2019)	5–9	COMB: 7.23 ± 1.42; CON: 7.06 ± 1.06	COMB: M = 7, F = 6; CON: M = 8, F = 8	DSM-IV-TR	Excluded: severe comorbid, neurological	15–19% on stimulants	Stable dose 1 month prior
Zhao et al. (2024)	6–12	INT: 8.5 (1.5), CON: 8.3 (1.1)	INT: F = 6, M = 34; CON: F = 11, M = 29	DSM-5	Excluded ASD, learning disabilities	None in past 6 months	Not allowed

RCT, randomised controlled trial; CT, controlled trial; RXT, randomised crossover trial; F, female; M, male; AET, aerobic exercise training; CET, cognitively engaging training; COT, coordinative training; COMB, combined training; MBT, mind–body training; ACC, attention control condition; WLC, waitlist control; CON, control; MPH, methylphenidate; DEX, dexamphetamine; NR, not reported; DSM, Diagnostic and Statistical Manual of Mental Disorders; ICD, International Classification of Diseases; DISC-IV, Diagnostic Interview Schedule for Children; K-SADS-PL, Kiddie Schedule for Affective Disorders and Schizophrenia-Present and Lifetime Version; ODD, oppositional defiant disorder; CD, conduct disorder; ASD, autism spectrum disorder; PDD, pervasive developmental disorder; SNAP-IV, Swanson, Nolan, and Pelham Rating Scale; CBCL, Child Behaviour Checklist.

Diagnoses of ADHD were made based on DSM-IV criteria (*n* = 7), DSM-5 criteria (*n* = 9), ICD-10 criteria (*n* = 1), or clinical diagnosis (*n* = 2), with several studies utilising structured diagnostic interviews, such as the DISC-IV or K-SADS-PL, to validate diagnoses (Table 1). The exclusion of comorbidity differed significantly; nine studies excluded neurological disorders, autism spectrum disorder, or other psychiatric disorders, whereas four studies explicitly included oppositional defiant disorder or conduct disorder (Table 1). Medication status showed a high degree of heterogeneity: seven studies (36.8%) demanded that the participants were medication-naive or medication-free during a defined period of washout, four studies (21.1%) included participants on stable medication regimens who continued to take medication during the study, five studies (26.3%) used mixed populations with different medication exposure, and three studies (15.8%) did not specify medication status (Table 1).

### 3.3 Intervention characteristics

There was substantial heterogeneity in exercise interventions by modality, dosage, and delivery parameters (Table 2). The intervention lasted between 4 and 78 weeks (median: 12 weeks), with the majority of the studies (9 studies) (47.3%) using programmes between 8 and 12 weeks. The frequency of sessions was between once a week and five sessions per week, with three sessions per week being the most prevalent (*n* = 10, 52.6%). The duration of individual sessions was 30–120 min (median: 60 min), leading to a total dose of 360–7,800 min of intervention. Intensity prescriptions, where reported, were mainly moderate-vigorous (60–80% heart rate maximum or heart rate reserve), though six studies (31.6%) did not specify intensity parameters (Table 2).

**Table 2:** Intervention characteristics of included studies.

Study ID	Arm	Intervention name/description	Duration (weeks)	Frequency (sessions/wk)	Session length (min)	Total dose (min)	Intensity	Setting
Benzing and Schmidt (2019)	CET vs. WLC	Xbox Kinect exergame (Shape UP): coordination, strength training with cognitive demands	8	3	30	720	Moderate-vigorous (75% max HR)	Home
Bustamante et al. (2016)	AET vs. ACC	After-school exercise: cooperative/competitive games, modified sports	10	5	60	3,000	Vigorous (75% max HR avg)	School gym
Chang et al. (2014)	COMB vs. WLC	Aquatic exercise: water aerobics (40 min) + perceptual-motor water exercises (40 min)	8	2	90	1,440	Moderate	Swimming pool
Chang et al. (2022)	COT vs. CON	Actual table tennis: basic skills, executive attention training, gaze pursuit/arm control	12	3	60	2,160	NR	School gym
Chang et al. (2022)	CET vs. CON	Nintendo Wii Sport table tennis exergame	12	3	60	2,160	NR	School/Home
Durgut et al. (2020)	COMB vs. AET	Treadmill running + ball exercises (throwing, catching, dribbling, coordination tasks)	8	3	60	1,440	Moderate-vigorous (NR%)	Hospital physiotherapy unit
Hattabi et al. (2022)	AET vs. WLT	Progressive swimming strokes: Freestyle, backstroke, breaststroke, butterfly; 60–80% HRmax maintained	12	3	60	2,160	Moderate-vigorous (60–80% HRmax)	Swimming pool
Huang et al. (2024)	COT vs. WLT	Progressive rope skipping: Weeks 1–2 unroped jumping; Weeks 3–4 single jump; Weeks 5–6 pad step; Weeks 7–8 tandem with timed relays	8	2	60	960	Moderate-vigorous (71% HRmax)	School gymnasium
Huang et al. (2025)	COT vs. WLT	20 inline skating skills taught progressively; Includes cognitive games (Simon Says, treasure hunt); Cardiovascular endurance training	12	2	80	1,920	Moderate-vigorous (NR%)	University gymnasium
Kadri et al. (2019)	MBT vs. ACC	Taekwondo (TKD): technical skill development (blocking, punching, kicking) and poomse (forms) with progressive difficulty	78	2	50	7,800	NR	Private martial arts Dojang
Liang et al. (2022)	COMB vs. WLT	Combined aerobic + neurocognitive exercise: stationary cycling, table tennis with robot (variable colours, directions, speeds requiring selective attention)	12	3	60	2,160	Moderate-vigorous (60–80% HRmax; avg. 75.97%)	NR
Liu et al. (2025)	AET vs. WLT	Aerobic exercise-based PA: enjoyable activities, mini games (hopscotch, obstacle running), coordination exercises, outdoor activities (hiking, cycling, rowing)	12	1	60	720	Progressive (light to moderate-vigorous)	School and community recreational facilities
Ludyga et al. (2022)	MBT vs. WLT	Judo training—basic techniques for attack, defence, injury prevention; techniques + playful fitness exercises + Randori	12	2	60	1,440	Moderate (RPE 13.3 ± 0.7)	University sports facility
Ludyga et al. (2023)	MBT vs. WLT	Judo training—techniques for attack/defence, playful fitness exercises, Randori (free fighting)	12	2	60	1,440	Moderate (RPE 13.3 ± 0.7)	NR
Memarmoghaddam et al. (2016)	COMB vs. CON	Selected exercise programme: aerobic warm-up (15 min), goal-directed exercises with rackets/balls/targeting (25 min), station training (10 min), treadmill running (15 min), ball games (15 min), cool-down (10 min)	8	3	90	2,160	Moderate-Vigorous (65–80% HRR)	University Sports Hall
Nejati (2021)	CET vs. AET	EXCIR—Exercise for Cognitive Improvement and Rehabilitation: 12 physical tasks with progressive cognitive demands at 10 levels (e.g., Colour Jumping, Arrow Jumping, Pattern Walking, Go/No-Go games)	4–5	3	40–50	480–750	NR	Research centre
Nejati and Derakhshan (2021)	CET vs. ACC	Cognitive-motor dual-task: balance exercises on wobble board with simultaneous N-back, Stroop, Go/No-Go tasks	4–5	3	40–50	1,440–2,250	NR	NR
Pan et al. (2016)	COT vs. WLT	Table tennis: warm-up, motor skills, EF training, group games, cooldown	12	2	70	1,680	Moderate	University table tennis centre
Smith et al. (2019)	COMB vs. CON	IBBS: Computerised cognitive training (brain), physical exercises (body), Good Behaviour Game (social)	15	3	120	5,400	Moderate	After-school
Zhao et al. (2024)	CET vs. WLT	BrainFit: Digital cognitive-physical training via iPad; AR-based gamified cognitive + movement training	4	3	30	360	Adaptive (game-based)	Hospital

AET, aerobic exercise training; CET, cognitively engaging training; COT, coordinative training; COMB, combined training; MBT, mind–body training; ACC, attention control condition; WLC, waitlist control; CON, control; HR, heart rate; HRmax, maximum heart rate; HRR, heart rate reserve; RPE, rating of perceived exertion; NR, not reported; AR, augmented reality; RA, research assistant.

Modes of exercise included cognitively engaging training [*n* = 5 (Chang et al., 2022; Nejati, 2021; Nejati and Derakhshan, 2021; Zhao et al., 2024; Benzing and Schmidt, 2019)], which involved exergaming, cognitive-motor dual-tasks, or digitally-enhanced activities; coordinative training [*n* = 4 (Huang et al., 2025; Chang et al., 2022; Pan et al., 2016; Huang et al., 2024)], which involved table tennis, rope skipping, and inline skating; combined exercise interventions [*n* = 5 (Memarmoghaddam et al., 2016; Chang et al., 2014; Liang et al., 2022; Smith et al., 2019; Durgut et al., 2020)]; mind–body exercises [*n* = 3 (Ludyga et al., 2023; Kadri et al., 2019; Ludyga et al., 2022)], which included taekwondo and judo; and aerobic exercise alone [*n* = 5 (Liu et al., 2025; Nejati and Derakhshan, 2021; Bustamante et al., 2016; Durgut et al., 2020; Hattabi et al., 2022)]. Many interventions were conducted in group formats (*n* = 14, 73.7%) versus individual (*n* = 4, 21.1%) or mixed (*n* = 1, 5.3%) formats. Intervention settings were school facilities (*n* = 7), university or research centres (*n* = 6), community facilities (*n* = 3), hospitals (*n* = 2), and home environments (*n* = 1). All interventions were characterised by direct oversight by qualified specialists such as physical education specialists, coaches, physiotherapists, or trained research assistants. Control conditions included waitlist controls (*n* = 13, 68.4%), attention controls involving sedentary activities (*n* = 4, 21.1%), or regular care (*n* = 2, 10.5%) (Table 2).

### 3.4 Risk of bias assessment

**Figure 2:**
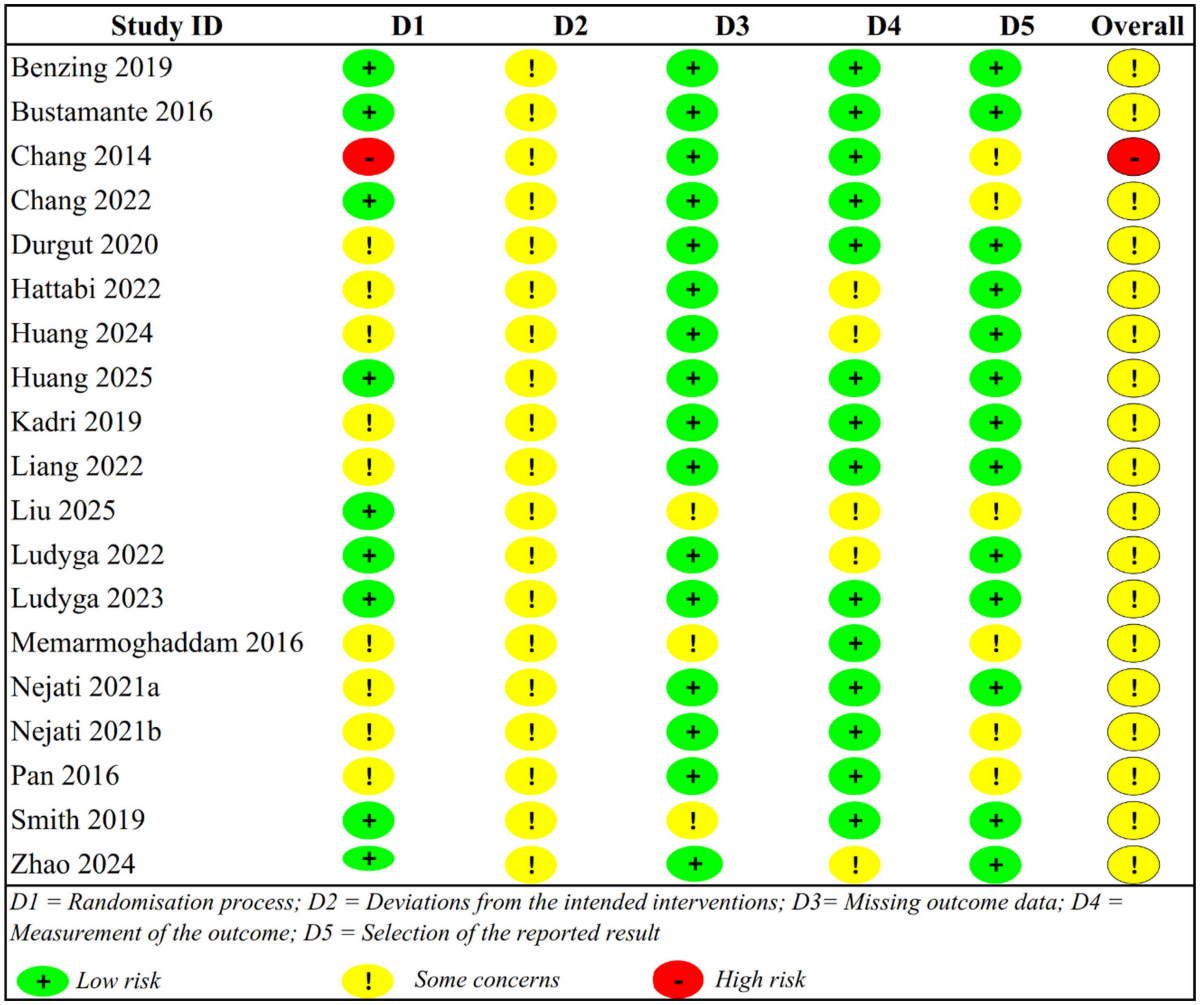
Study-level risk of bias assessment across all domains.

**Figure 3:**
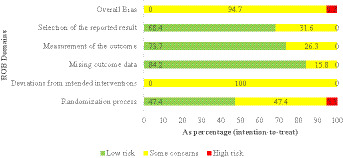
Risk of bias summary.

Assessment of risk of bias showed that the methodology was problematic in two domains, with only one study (5.3%) demonstrating an overall high risk of bias (Figures 2, 3). The quality of randomisation was mixed, with 47.4% of studies having low-risk, 47.4% raising some concern because of poor reporting of the allocation concealment methods, and 1 study (Chang et al., 2014) scoring high risk because of poor reporting of the allocation concealment methods. Concerns about non-adherence to intended interventions were present in all studies and are inherent to blinding participants and intervention providers in exercise studies, and the possibility of a performance bias between different motivation or expectation effects. There were no significant issues with missing outcome data, and 84.2% of studies had a low risk because sufficient retention and intention-to-treat were met (Figures 2, 3).

There was a significant difference in outcome measurement bias; 73.7% exhibited low risk with blinded assessment or objective performance-based scales, whereas 26.3% expressed concern with subjective rating scales filled in by unblinded raters prone to expectation bias. Selective reporting predominantly featured low risk (68.4), but 31.6% expressed concerns about missing full outcome reporting or non-adherence to pre-specified analyses (Figures 2, 3). The high risk of bias across all studies (94.7%) was largely driven by matters of universal importance in Domain 2 (deviations of intended interventions), and indicated that there were systematic issues with conducting sufficiently blinded exercise intervention trials in paediatric ADHD populations despite otherwise sound methodological practices.

### 3.5 Network meta-analysis

#### 3.5.1 Network geometry: inhibitory control accuracy

**Figure 4:**
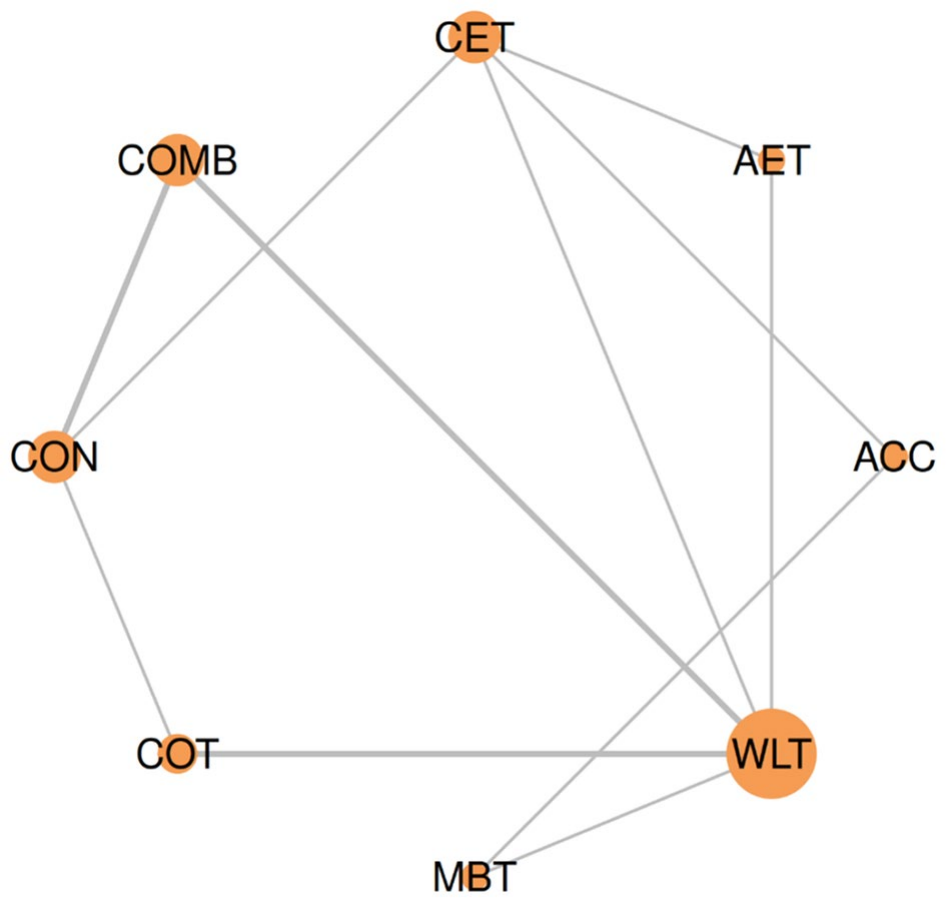
Network geometry for inhibitory control accuracy outcome.

The network comprised 8 treatment nodes and 11 direct comparisons (Figure 4). WLT served as the most connected node with five direct comparisons, followed by CON and CET with three connections each. COMB demonstrated dual connectivity to both active (CON) and passive (WLT) controls. The network geometry revealed two distinct clusters: exercise interventions (CET, COMB, COT) predominantly compared against passive waitlist controls, while CET uniquely bridged to active comparators (AET, ACC). MBT and AET showed limited connectivity with single pathways to WLT and ACC, respectively. No closed loops existed, indicating all indirect comparisons relied entirely on single connecting pathways.

#### 3.5.2 Network meta-analysis results: inhibitory control accuracy

##### 3.5.2.1 League table results

The league table reports pairwise standardised mean differences (SMDs) of all treatment comparisons, with direct evidence reported in the upper triangle and mixed evidence (combining direct and indirect comparisons) in the lower triangle (Table 3). Out of the 28 possible pairwise comparisons, 11 were informed by direct evidence of head-to-head trials, with the remaining 17 estimated by indirect comparisons using connecting pathways in the network. The coordinative training proved to be statistically significantly better than various comparators, becoming the most effective intervention. Direct evidence indicated that COT had a significant advantage over waitlist control (SMD = 2.21, 95% CI: 1.14–3.27), which is a large effect in favour of exercise (Table 3). This result suggests that children who underwent coordinative training showed significantly higher gains in the accuracy of inhibitory control in comparison to those who awaited intervention. COT also demonstrated notable benefits over attention control in direct comparison (SMD = −2.12, 95% CI: −3.51 to −0.72), indicating that the benefits of coordinative exercise are not solely due to placebo or attention benefits of engaging in structured activities.

**Table 3:** Network meta-analysis for all outcomes.

Interventions	ACC	AET	CET	COMB	CON	COT	MBT	WLT
ACC	ACC	–	−0.57 (−1.95 to 0.81)	–	–	–	−2.12 (−3.51 to −0.72)	–
AET	−0.64 (−2.10 to 0.82)	AET	−0.84 (−2.22 to 0.54)	–	–	–	–	−0.08 (−1.33 to 1.17)
CET	−1.13 (−2.28 to 0.02)	−0.49 (−1.52 to 0.54)	CET	–	0.45 (−0.91 to 1.80)	–	–	0.02 (−1.22 to 1.26)
COMB	−1.48 (−2.89 to −0.06)	−0.83 (−2.06 to 0.40)	−0.34 (−1.38 to 0.69)	COMB	0.66 (−0.31 to 1.62)	–	–	0.28 (−0.65 to 1.21)
CON	−1.02 (−2.43 to 0.39)	−0.37 (−1.62 to 0.88)	0.12 (−0.86 to 1.10)	0.46 (−0.36 to 1.27)	CON	−1.03 (−2.41 to 0.34)	–	–
COT	−2.78 (−4.27 to −1.29)	−2.14 (−3.45 to −0.82)	−1.65 (−2.79 to −0.51)	−1.31 (−2.38 to −0.23)	−1.77 (−2.78 to −0.76)	COT	–	2.21 (1.14 to 3.27)
MBT	−1.54 (−2.70 to −0.38)	−0.90 (−2.32 to 0.52)	−0.41 (−1.62 to 0.81)	−0.07 (−1.39 to 1.26)	−0.52 (−1.88 to 0.83)	1.24 (−0.16 to 2.64)	MBT	0.04 (−1.23 to 1.32)
WLT	−1.01 (−2.25 to 0.22)	−0.37 (−1.37 to 0.63)	0.12 (−0.71 to 0.95)	0.46 (−0.33 to 1.25)	0.00 (−0.89 to 0.89)	1.77 (0.86 to 2.67)	0.53 (−0.57 to 1.62)	WLT

Comparisons of mixed evidence, using direct and indirect paths, found that COT performed significantly better than any other treatment within the network. In particular, COT showed notable superiority over ACC (SMD = −2.78, 95% CI: −4.27 to −1.29), AET (SMD = −2.14, 95% CI: −3.45 to −0.82), CET (SMD = −1.65, 95% CI: −2.79 to −0.51), COMB (SMD = −1.31, 95% CI: −2.38 to −0.23). These repeated results with active and passive comparators reinforce the belief in the efficacy of coordinative training in inhibitory control (Table 3).

Combined exercise showed a strong effect relative to attention control (COMB vs. ACC: SMD = −1.48, 95% CI: −2.89 to −0.06), indicating that multimodal exercise programmes that include multiple training elements can be beneficial compared with non-exercise training. Mind–body training was also significantly superior to attention control (MBT vs. ACC: SMD = −1.54, 95% CI: −2.70 to −0.38), meaning that contemplative movement practices, including yoga and tai chi, have significant benefits on inhibitory control that are not matched by attention-matched comparators. Nevertheless, comparisons with other active exercise modalities showed no significant differences, indicating similar efficacy of non-coordinative interventions. The SMD between CET and AET was −0.49 (95% CI: −1.52 to 0.54), showing no significant difference between cognitively engaging and aerobic exercise. CET vs. CON showed an SMD of 0.12 (95% CI: −0.86 to 1.10) and CET vs. WLT showed an SMD of 0.02 (95% CI: −1.22 to 1.26), neither significant. COMB demonstrated non-significant effects relative to passive controls (COMB vs. WLT: SMD = 0.28, 95% CI: −0.65 to 1.21; COMB vs. CON: SMD = 0.66, 95% CI: −0.31 to 1.62) (Table 3), indicating that combined strategies do not appear to provide an incremental benefit relative to single-modality interventions in comparison to control conditions.

##### 3.5.2.2 Treatment rankings

P-scores and surface under the cumulative ranking curve (SUCRA) probabilities provided a probabilistic ordering of treatment efficacy for inhibitory control accuracy (Table 4). These rankings should be interpreted as estimates of the relative probability of superiority under the current evidence base, not as definitive hierarchies; in a sparse network with no closed loops and moderate-to-substantial between-study heterogeneity (*I*^2^ = 74.7%), rankings are sensitive to the addition or removal of individual studies and carry meaningful uncertainty.

**Table 4:** SUCRA treatment results.

Treatment	P-score	Rank mean	Rank
COT	0.9924	0.99	1
COMB	0.691	0.69	2
MBT	0.6877	0.69	2
WLT	0.5023	0.5	4
CON	0.4155	0.42	5
CET	0.4147	0.41	6
ACC	0.2423	0.24	7
AET	0.0541	0.05	8

With that caveat, coordinative training ranked highest (P-score = 0.99), suggesting a high probability of being the most effective modality in the current network. Combined exercise and mind–body training ranked second and third, respectively (both P-score = 0.69), indicating broadly comparable and intermediate effectiveness. Cognitively engaging training ranked fourth (P-score = 0.50), placing it near the centre of the treatment hierarchy. Passive control conditions, waitlist (P-score = 0.41) and no-intervention control (P-score = 0.42), occupied similar mid-to-lower positions, while aerobic exercise training ranked seventh (P-score = 0.24) and attention control ranked last (P-score = 0.05). The gap between coordinative training and all other active interventions is notable, though the wide prediction intervals reported in Table 5, particularly the upper bound of COT’s interval approaching zero (PI: −3.62 to 0.09), indicate that effect sizes in future studies may be smaller than the current estimate suggests. Rankings for all other interventions had prediction intervals spanning zero, indicating that the direction of effect remains uncertain in future study conditions.

**Table 5:** Prediction intervals summary.

Treatment	Point estimate	CI lower	CI upper	PI lower	PI upper	CI width	PI width	Width ratio
ACC	1.02	−0.39	2.43	−1.19	3.22	2.82	4.41	1.56
AET	0.37	−0.88	1.62	−1.69	2.43	2.50	4.12	1.65
CET	−0.12	−1.10	0.86	−1.95	1.72	1.96	3.67	1.87
COMB	−0.46	−1.27	0.36	−2.17	1.25	1.63	3.42	2.10
COT	−1.77	−2.78	−0.76	−3.62	0.09	2.02	3.71	1.84
MBT	−0.53	−1.88	0.83	−2.68	1.63	2.72	4.31	1.59
WLT	0.00	−0.89	0.89	−1.77	1.77	1.79	3.53	1.98

##### 3.5.2.3 Heterogeneity assessment

Statistical heterogeneity (moderate to substantial) was observed across the network. The between-study standard deviation (*τ*) was 0.35, and the within-study standard deviation (*τ*) was 0.59. The *I*^2^ value of 74.70% shows that about three-quarters of the observed variance is due not to sampling error but to true heterogeneity. The *Q* test by Cochran revealed substantial heterogeneity (*Q* = 27.66, df = 7, *p* < 0.001). This degree of heterogeneity, although significant, is not surprising when considering the clinical heterogeneity of the studies incorporated, such as differences in intervention regimens and dosages, ages of participants, ADHD severity, and measures of outcome.

Compared to confidence intervals, prediction intervals that consider heterogeneity in projecting treatment effects in future studies were broader across all treatments (Table 5). The width ratios were 1.56 on ACC and 2.10 on COMB, meaning that prediction intervals were about 1.5–2 times broader than the confidence intervals. This expansion indicates uncertainty regarding the effects of treatment in future clinical environments that might not be consistent with the studies included. Most remarkably, COT had a negative upper prediction interval bound (PI: −3.62 to 0.09), indicating that even with the existing heterogeneity, coordinative training would most likely show advantages in most future study conditions. The upper limit to zero, however, is worth interpreting cautiously, since there are settings that will experience negligible effects. The other treatments all had prediction intervals spanning across zero, suggesting that their effects may be both positive and negative depending on the study-specific factors.

Sensitivity analyses between fixed and random effects models (Table 6) demonstrated no significant discrepancies among treatments, with variations ranging between −0.02 (COT) and 0.25 (AET). This consistency implies that the treatment effects estimates are robust in the presence of the observed heterogeneity, and the conclusions are consistent across modelling assumptions.

**Table 6:** Fixed vs. random sensitivity analysis.

Treatment	Random effect	Common effect	Difference
ACC	1.02	0.95	0.06
AET	0.37	0.12	0.25
CET	−0.12	−0.22	0.11
COMB	−0.46	−0.44	−0.02
COT	−1.77	−1.74	−0.02
MBT	−0.53	−0.51	−0.02
WLT	0.00	−0.16	0.16

##### 3.5.2.4 Publication bias

Egger regression test revealed that there was no significant asymmetry in the funnel plot (test statistic = 0.76, *p* = 0.460), and no evidence of publication bias was found statistically. This observation suggests that selective reporting of positive results or non-publication of studies with null results are unlikely to have a significant effect on the network meta-analysis results.

##### 3.5.2.5 Consistency assessment

The back-calculation of the Separating Indirect from Direct Evidence (SIDE) analysis indicated that no significant disparity existed between direct and indirect evidence in any comparison (all *p* > 0.05; Table 7). The greatest absolute changes were between ACC and MBT (direct: −2.12; indirect: −0.26; difference = −1.85, *p* = 0.146) and COT and CON (direct: 1.03; indirect: 2.63; difference = −1.60, *p* = 0.122). Although these numerical disparities imply a degree of variation between direct and indirect estimates, statistical tests indicated consistency across the network. The lack of major inconsistency (all *p*-values between 0.122 and 0.828) confirms the validity of combining direct and indirect evidence to estimate treatment effects and demonstrates that the transitivity assumption underlying network meta-analysis was not broken.

**Table 7:** The separating indirect from direct evidence analysis.

Comparison	*k*	Prop	NMA	Direct	Indirect	Diff	*z*	*p*-value
ACC vs. CET	1	0.7	−1.133	−0.570	−2.424	1.854	1.450	0.146
ACC vs. MBT	1	0.69	−1.540	−2.118	−0.264	−1.854	−1.450	0.146
AET vs. CET	1	0.56	−0.490	−0.842	−0.042	−0.799	−0.750	0.452
AET vs. WLT	1	0.64	−0.372	−0.082	−0.881	0.799	0.750	0.452
CET vs. CON	1	0.52	0.117	0.446	−0.243	0.689	0.690	0.492
CET vs. WLT	1	0.45	0.118	0.016	0.201	−0.185	−0.220	0.828
COMB vs. CON	2	0.71	0.460	0.656	−0.024	0.680	0.740	0.458
COMB vs. WLT	2	0.73	0.461	0.279	0.959	−0.680	−0.740	0.458
COT vs. CON	1	0.54	1.767	1.031	2.626	−1.596	−1.540	0.122
COT vs. WLT	2	0.73	1.768	2.206	0.610	1.596	1.540	0.122
MBT vs. WLT	1	0.74	0.526	0.045	1.899	−1.854	−1.450	0.146

Comparison, Treatment comparison; *k*, Number of studies providing direct evidence; prop, Direct evidence proportion; NMA, Estimated treatment effect (SMD) in network meta-analysis; direct, Estimated treatment effect (SMD) derived from direct evidence; indirect, Estimated treatment effect (SMD) derived from indirect evidence; Diff, Difference between direct and indirect treatment estimates; *z*, *z*-value of test for disagreement (direct versus indirect); *p*-value, *p*-value of test for disagreement (direct versus indirect).

##### 3.5.2.6 Certainty of evidence

Certainty of evidence was assessed using the GRADE framework adapted for network meta-analysis (Cochrane Training, 2023; Brignardello-Petersen et al., 2019) (Table 8). All comparisons were downgraded at least one level for serious risk of bias, reflecting the inherent impossibility of blinding participants and personnel to exercise intervention allocation across all included trials. Coordinative training (COT) was rated as moderate certainty evidence for inhibitory control accuracy. Although the pooled estimate is large and statistically significant (SMD = 1.78, 95% CI: 0.72–2.84), the rating was not further downgraded for imprecision given that the confidence interval excludes the null and the estimate is based on three RCTs with a combined sample of 88 participants, approaching, though not fully meeting, optimal information size. The estimate was not downgraded for indirectness as the COT-versus-waitlist comparison is supported by direct evidence.

**Table 8:** Summary of findings.

Outcomes	Anticipated absolute effects^*^ (95% CI)	No. of participants (studies)	Certainty of the evidence (GRADE)
	Risk with IC accuracy		
AET	SMD **0.08 lower** (0.55 lower to 0.39 higher)	69 (1 RCT)	⨁⨁◯◯ Low^a,b^
CET	SMD **0.39 higher** (0.01 lower to 0.78 higher)	171 (4 RCTs)	⨁⨁◯◯ Low^c,d^
COMB	SMD **0.45 higher** (0.12 lower to 1.02 higher)	170 (4 RCTs)	⨁⨁◯◯ Low^e,f^
COT	SMD **1.78 higher** (0.72 higher to 2.84 higher)	88 (3 RCTs)	⨁⨁⨁◯ Moderate^g^
MBT	SMD **2.44 higher** (1.84 higher to 3.04 higher)	96 (2 RCTs)	⨁⨁⨁◯ Moderate^h^

* The risk in the intervention group (and its 95% confidence interval) is based on the assumed risk in the comparison group and the relative effect of the intervention (and its 95% CI).

CI, confidence interval; SMD, standardised mean difference.

GRADE Working Group grades of evidence.

High certainty: We are very confident that the true effect lies close to that of the estimate of the effect.

Moderate certainty: we are moderately confident in the effect estimate: the true effect is likely to be close to the estimate of the effect, but there is a possibility that it is substantially different.

Low certainty: our confidence in the effect estimate is limited: the true effect may be substantially different from the estimate of the effect.

Very low certainty: we have very little confidence in the effect estimate: the true effect is likely to be substantially different from the estimate of effect.

Explanations.

^a^Single RCT with inability to blind participants and personnel to exercise allocation; outcome assessors may have been aware of group assignment.

^b^Wide confidence interval crossing null effect and clinically important thresholds; very small sample size (*n* = 69) from single RCT fails to meet optimal information size.

^c^Blinding of participants and personnel not feasible across all 4 RCTs; potential performance bias due to differential expectations between exergaming/table tennis and control conditions.

^d^Confidence interval crosses null effect (0.01 lower to 0.78 higher); total sample size of 171 participants approaches but may not meet optimal information size for detecting small-to-moderate effects.

^e^Inability to blind participants to multimodal exercise programmes across 4 RCTs; variable quality in allocation concealment and outcome assessment blinding.

^f^Confidence interval includes null effect (0.12 lower to 1.02 higher); cannot exclude trivial effect or moderate benefit; 170 participants insufficient for precise estimation.

^g^Blinding of participants impossible for coordinative exercise interventions across 3 RCTs; some studies had unclear allocation concealment; outcome assessors not consistently blinded.

^h^Yoga and tai chi interventions cannot be blinded in 2 RCTs; participants aware of group allocation may have differential expectations affecting performance on cognitive tasks.

Bold values indicate statistically significant results (*p* < 0.05) or the highest/best performing intervention in the corresponding comparison.

Mind–body training (MBT) was also rated as moderate certainty. The MBT estimate (SMD = 2.44, 95% CI: 1.84–3.04) is based on only two RCTs (*n* = 96) and relies on a single indirect pathway through waitlist control for comparisons with other active interventions. A downgrade for imprecision was considered, given the small sample size, but was ultimately not applied, given the precision of the confidence interval. Clinicians should nonetheless note that the evidence base for MBT is the thinnest among the moderately-rated modalities. Cognitively engaging training (CET), combined exercise (COMB), and aerobic exercise training (AET) were all rated as low certainty, downgraded for both serious risk of bias and serious imprecision; confidence intervals crossed the null effect for all three, and sample sizes were insufficient to resolve whether true effects exist. The single-study evidence base for AET (*n* = 69) was particularly limited. These ratings reflect genuine uncertainty about the effectiveness of these modalities for inhibitory control accuracy and should not be interpreted as evidence of ineffectiveness.

##### 3.5.2.7 Subgroup analysis

###### 3.5.2.7.1 Age range

**Figure 5:**
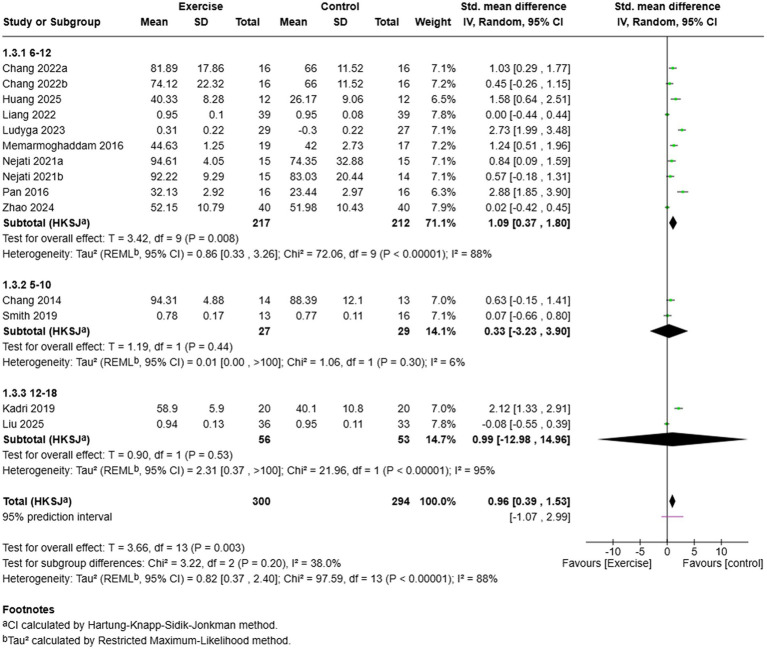
Subgroup analysis based on age range.

Age subgroup analysis showed varying effects at different developmental stages (Figure 5). A large moderate-to-large effect in favour of exercise was observed in children aged 6–12 years (SMD = 1.09, 95% CI: 0.37–1.80, *k* = 10), with a substantial heterogeneity (*I*^2^ = 88%). The 5–10 years subgroup demonstrated a non-significant small effect (SMD = 0.33, 95% CI: −3.23 to 3.90, *k* = 2) with low heterogeneity (*I*^2^ = 6%). There was a non-significant effect in adolescents aged 12–18 years (SMD = 0.99, 95% CI: −12.98 to 14.96, *k* = 2) with high heterogeneity (*I*^2^ = 95%). The sub-group difference test was not significant (*χ*^2^ = 3.22, df = 2, *p* = 0.20, *I*^2^ = 38.0), which means that the effect modification by age is statistically non-significant. The sustained meaningful effect in the 6–12 years subgroup, however, indicates that school-age children might reap the most benefits of exercise interventions on inhibitory control.

###### 3.5.2.7.2 Dosage of exercise

**Figure 6:**
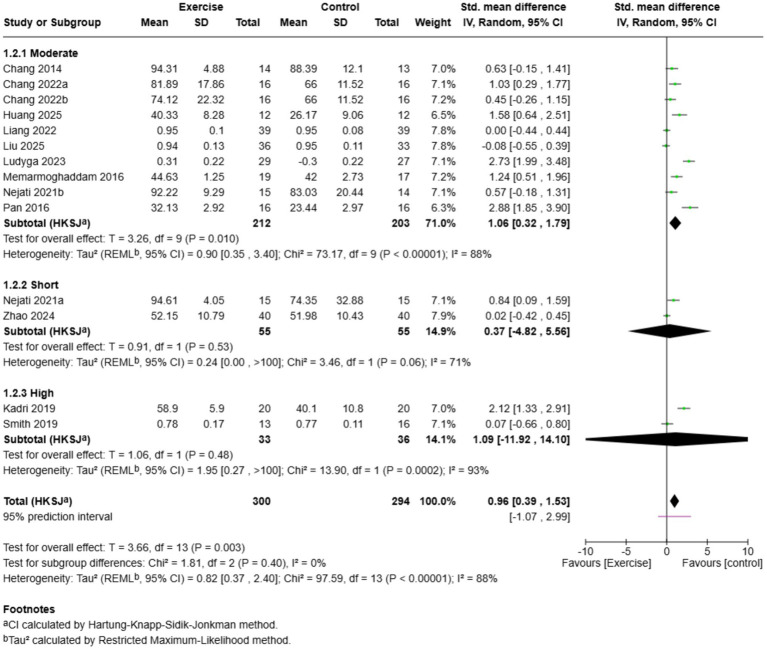
Subgroup analysis based on exercise total dosage.

Subgroup analysis based on total exercise dosage (frequency x duration x session length in minutes) showed different effects among dosage groups (Figure 6). Interventions based on moderate dosage (481–2,880 min) showed a significant effect in favour of exercise (SMD = 1.06, 95% CI: 0.32–1.79, *k* = 10) and high heterogeneity (*I*^2^ = 88%). There was no significant effect of short dosage programmes (<480 min) (SMD = 0.37, 95% CI: −4.82 to 5.56, *k* = 2, *I*^2^ = 71%). Interventions with high dosage (>2,880 min) also showed no significant effect (SMD = 1.09, 95% CI: −11.92 to 14.10, *k* = 2, *I*^2^ = 93%). Subgroup difference test was non-significant (2 = 1.81, df = 2, *p* = 0.40, *I*^2^ = 0%), indicating no apparent dose–response relationship. Nonetheless, the strongest evidence of benefit was observed with moderate dosage interventions, indicating that programmes providing 481–2,880 total minutes of exercise can potentially optimise improvements in inhibitory control in children with ADHD.

### 3.6 Pairwise meta-analysis results

#### 3.6.1 Inhibitory control reaction time

**Figure 7:**
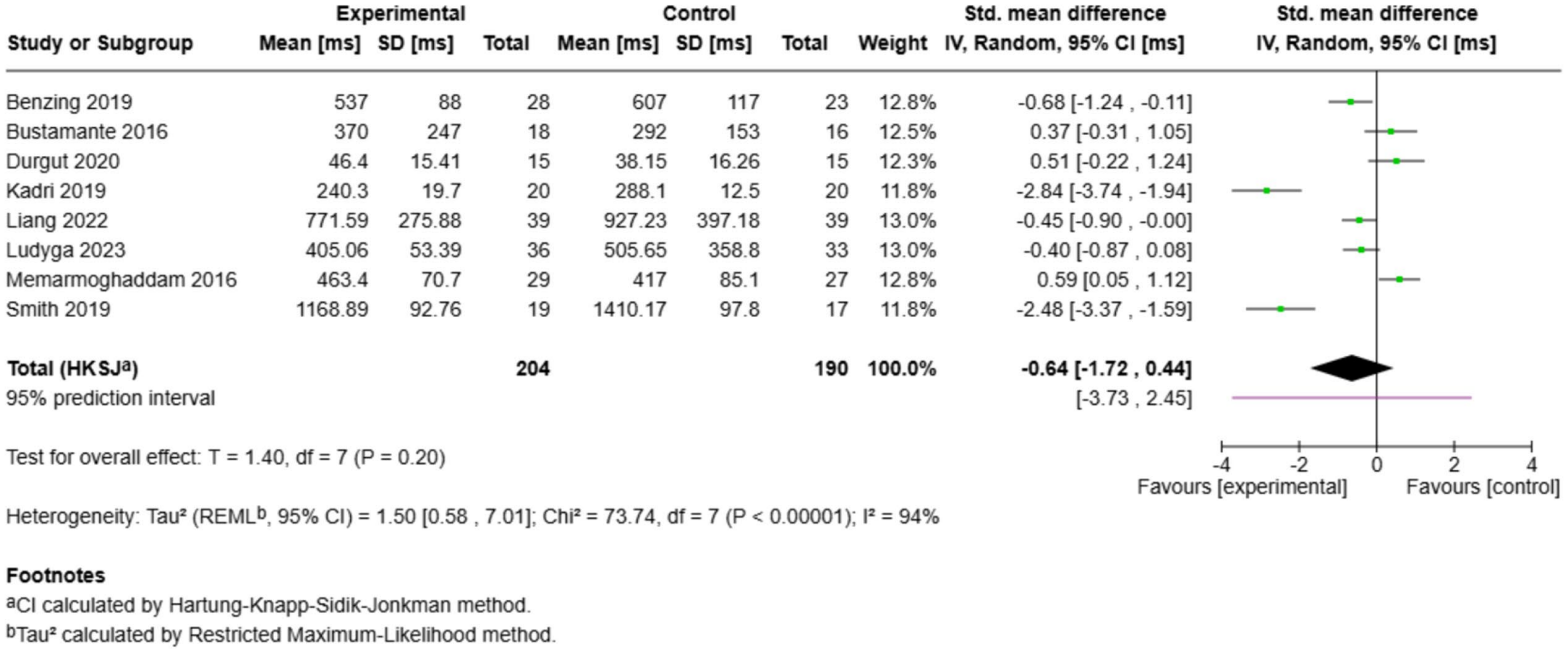
Pairwise meta-analysis results of inhibitory control reaction time outcome.

Eight studies investigated the impact of exercise on inhibitory control reaction time (Figure 7). The combined effect was insignificant (SMD = −0.64, 95% CI: −1.72 to 0.44, *p* = 0.20) and heterogeneity was large (*I*^2^ = 94%). Individual study effects were also widely different, with the strongest influence benefiting exercise [Kadri et al. Kadri et al., 2019: SMD = −2.84; Smith et al., 2019: SMD = −2.48] and the smallest effects benefiting control [Memarmoghaddam et al., 2016: SMD = 0.59; Bustamante et al., 2016: SMD = 0.37]. The large range of prediction (−3.73, 2.45) reflects significant uncertainty concerning future study effects. Although certain studies have shown considerable decreases in reaction time, the lack of consistent direction of effects across studies prevents conclusive results regarding the effectiveness of exercise in enhancing the speed of inhibitory control processing in children with ADHD.

#### 3.6.2 Working memory—accuracy

**Figure 8:**
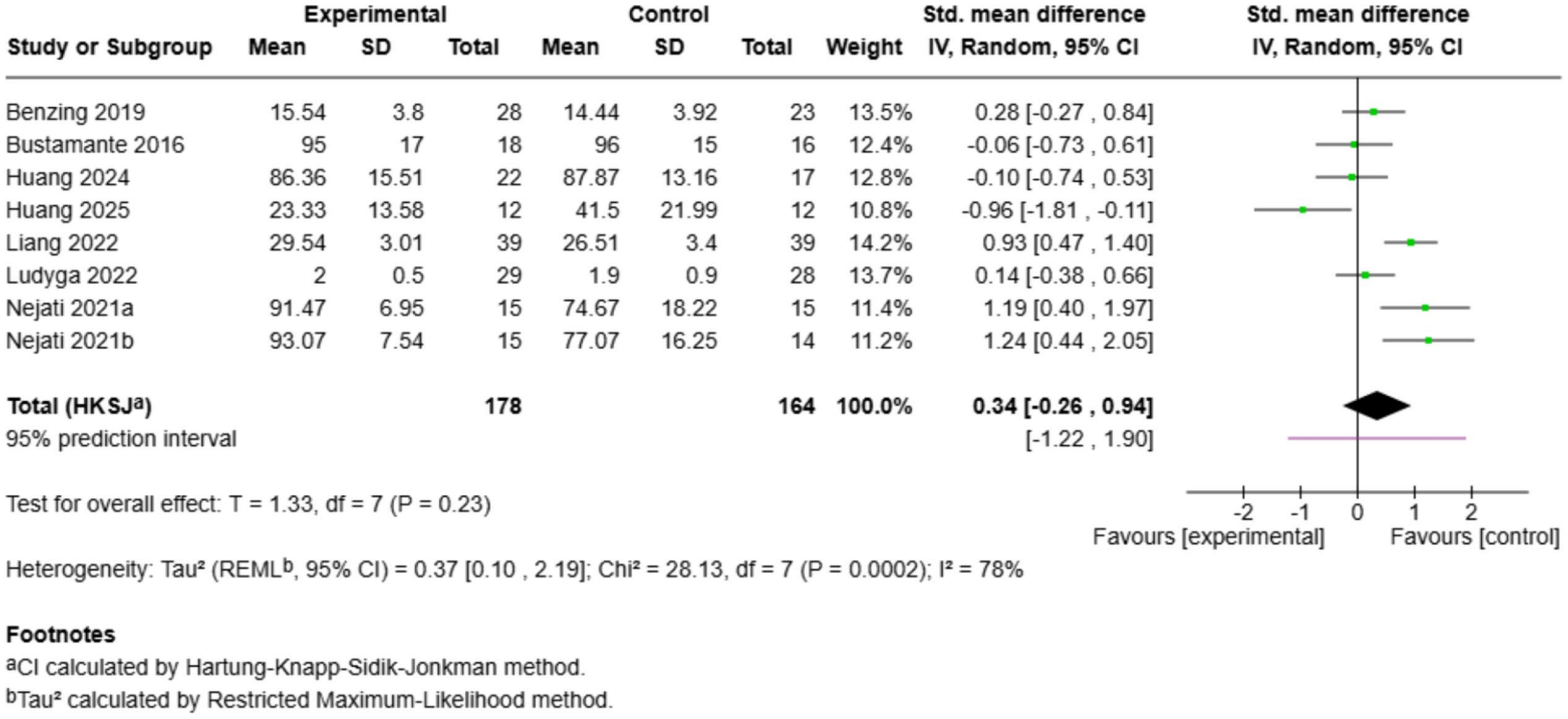
Pairwise meta-analysis results of working memory accuracy outcome.

Eight studies evaluated the impact of exercise on working memory accuracy (Figure 8). The overall effect was not significant (SMD = 0.34, 95% CI: −0.26 to 0.94, *p* = 0.23), and the heterogeneity was high (*I*^2^ = 78%). The effects were moderate [Nejati, 2021: SMD = 1.19; Nejati and Derakhshan, 2021: SMD = 1.24; Liang et al., 2022: SMD = 0.93] or negative [Huang et al., 2025: SMD = −0.96]. A prediction interval that cuts across zero (−1.22 to 1.90) shows a lot of uncertainty. Although Memarmoghaddam et al. (2016) and Nejati (2021) repeatedly reported advantages of cognitively engaging training, other studies, including Liu et al. (2025) (SMD = −0.06), Ludyga et al. (2022) (SMD = 0.14), and Zhao et al. (2024) (SMD = −0.10), revealed minimal effects. The inconsistent results indicate that the effects of exercise on working memory can be contingent on the type of intervention or the characteristics of the participants that are not accounted for in the analysis.

#### 3.6.3 Cognitive flexibility—accuracy

**Figure 9:**
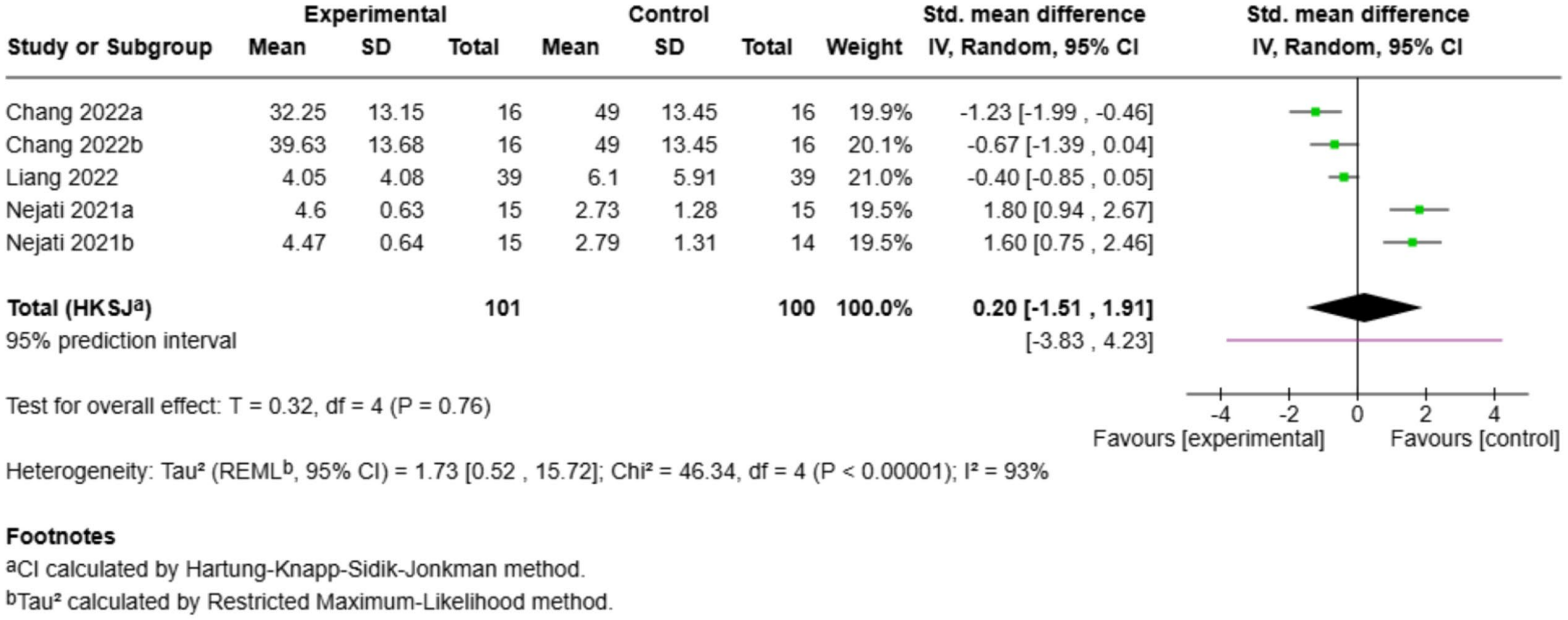
Pairwise meta-analysis results of cognitive flexibility accuracy outcome.

Out of the 19 included studies, five investigated the impact of exercise on cognitive flexibility accuracy (Figure 9). The overall effect was insignificant (SMD = 0.20, 95% CI: −1.51 to 1.91, *p* = 0.76), and there was significant heterogeneity (*I*^2^ = 93%). Effects varied considerably across studies: Memarmoghaddam et al. (2016) (SMD = 1.80) and Nejati (2021) (SMD = 1.60) reported substantially larger improvements with cognitively engaging training, whereas Chang et al. (2022) (SMD = −1.23) and Chang et al. (2022) (SMD = −0.67) found outcomes favouring control conditions. Liang et al. (2022) demonstrated a small non-significant effect (SMD = −0.40). The uncertainty surrounding similar WCST-based studies and the inconsistency of findings indicate that the methodology or population may have had differences affecting results, and it cannot be concluded with confidence that exercise is effective in cognitive flexibility (Lei et al., 2022; Tseng et al., 2022).

#### 3.6.4 ADHD symptoms (secondary outcome)

**Figure 10:**
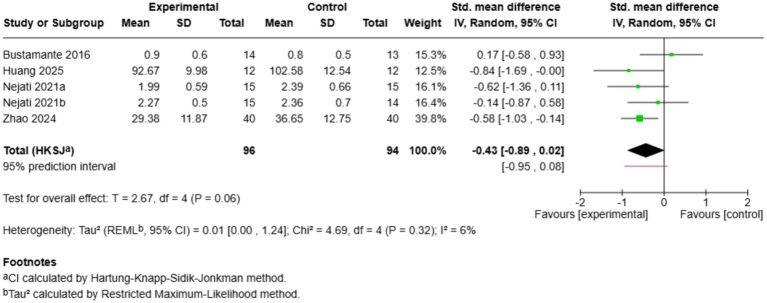
Pairwise meta-analysis results of parent-rated ADHD symptoms.

Five studies evaluated parent-rated ADHD symptoms (Figure 10). The overall effect was significant in favour of exercise (SMD = −0.43, 95% CI: −0.89 to 0.02, *p* = 0.06), with low heterogeneity (*I*^2^ = 6%). Zhao et al. (2024) (SMD = −0.58) and Huang et al. (2025) (SMD = −0.84) reported significant symptom improvements, whereas Nejati (2021) (SMD = −0.62) indicated a trend towards cognitively engaging training. Nejati and Derakhshan (2021) (SMD = −0.14) and Bustamante et al. (2016) (SMD = 0.17) reported small effects. The prediction interval (−0.95 to 0.08) is narrow, indicating rather stable effect sizes across settings. The overall effect was not statistically significant; however, the trend was consistently in favour of exercise, and low heterogeneity indicates that exercise interventions might be associated with small improvements in parent-reported ADHD symptoms, which should be explored further in larger samples.

#### 3.6.5 Motor performance (secondary outcome)

**Figure 11:**
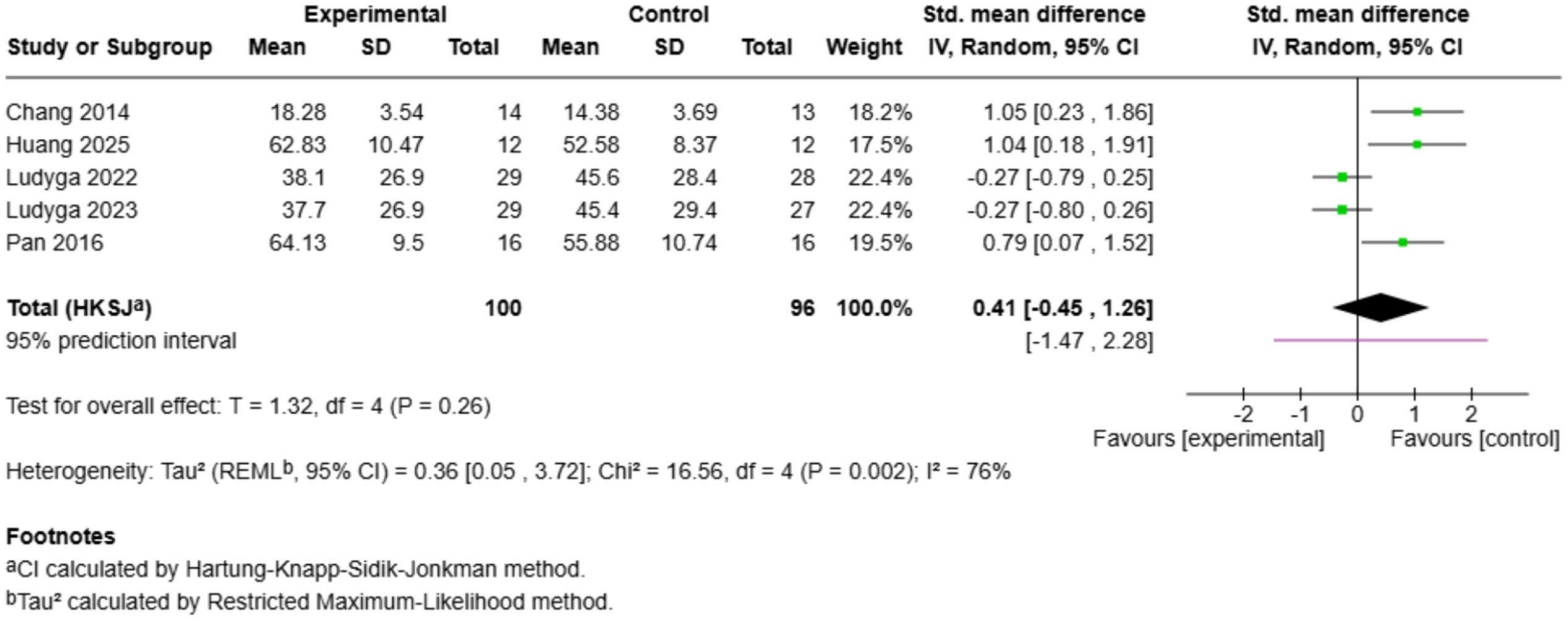
Pairwise meta-analysis results of motor performance outcome.

Five studies compared the effects of exercise on standardised motor measures (Figure 11). The overall effect was not significant (SMD = 0.41, 95% CI: −0.45 to 1.26, *p* = 0.26), and the heterogeneity was high (*I*^2^ = 76%). Chang et al. (2014) (SMD = 1.05), Huang et al. (2025) (SMD = 1.04), and Pan et al. (2016) (SMD = 0.79) reported significant increases in motor performance, whereas Ludyga et al. (2022) (SMD = −0.27) and Ludyga et al. (2023) (SMD = −0.27) exhibited small negative changes. The prediction interval (−1.47 to 2.28) demonstrates a good amount of doubt. The conflicting results could suggest disparities in exercise modes: coordinative training modalities (Chang, Huang, Pan) were always beneficial, and mind–body training modalities (Ludyga) did not indicate any advantage over control. This trend indicates that the benefits of motor performance improvements might be specific to one modality, with coordinative exercise offering more advantages than contemplative movement protocols.

## 4 Discussion

### 4.1 Principal findings and network coherence

This network meta-analysis synthesised evidence from 19 randomised controlled trials investigating the comparative effectiveness of various exercise modalities on executive function in children with ADHD. The most notable result was that coordinative training had a superior influence in enhancing inhibitory control accuracy, with an effect size (SMD = 1.77) that was significantly greater than other interventions and significantly higher than all comparators, such as aerobic exercise, cognitively engaging training, and combined exercise methods. By directly comparing exercise modalities through a network meta-analytic approach, this review fills a critical evidence gap: the existing literature has predominantly compared exercise versus no-exercise rather than evaluating which specific modality is most effective (Zhu et al., 2023; Tao et al., 2025). The findings provide the most granular comparative evidence to date on exercise modality selection for inhibitory control in paediatric ADHD, with direct clinical implications for families and practitioners seeking to prioritise specific activity types.

The observation that coordinative training showed greater effects is consistent with theoretical models that exercise involving dynamic environmental adaptation and complex motor-cognitive integration could have special advantages in executive function development (Zhu et al., 2023). Table tennis, rope skipping, and inline skating require quick processing of external stimuli, anticipatory planning, and ongoing regulation of motor responses-cognitive tasks that closely resemble attentional control and cognitive flexibility impairments typical of ADHD. This is consistent with the fact that various exercise modalities engage various executive function domains, as observed by Logan and Lim (2025), indicating that the cognitive load of coordinative activities can have more direct transfer effects to executive function than simple aerobic exercise. The limited number of studies that investigate coordinative training (*k* = 4) and the lack of closed loops in the network, however, make interpretation of this superiority cautious.

### 4.2 Inconsistent effects across executive function domains

The inconsistent results in the domains of executive functions pose a serious challenge to the belief in homogeneous cognitive gains of exercise. Although coordinative training demonstrated strong effects on inhibitory control accuracy, the computed network meta-analysis was not possible in working memory or cognitive flexibility because of the inadequacy of the studies and the paucity of network interconnections. The pairwise meta-analyses showed non-significant overall effects of working memory accuracy (SMD = 0.34, *p* = 0.23), inhibitory control reaction time (SMD = −0.64, *p* = 0.20), and cognitive flexibility (SMD = 0.20, *p* = 0.76), with large heterogeneity exceeding 75% in each case. This domain-specific variability challenges the hypothesis that exercise globally enhances executive functioning and indicates that various cognitive processes may vary in their responses to exercise interventions (Verburgh et al., 2014; Wang et al., 2024; Contreras-Osorio et al., 2021). This observation is especially significant due to the points made by Logan and Lim (2025), who noted that when developing interventions, one must not only take into account the cognitive domain but also exercise dose and not treat ADHD or executive functioning as monolithic constructs.

The extreme heterogeneity observed across pairwise meta-analyses (*I*^2^ = 78–94%) demands careful interpretation. In domains where heterogeneity is this high, the pooled SMD has limited practical meaning as a summary estimate; the prediction intervals, which project expected effects in future individual studies, are the more clinically informative statistic. The prediction intervals for inhibitory control reaction time (−3.73 to 2.45), working memory accuracy (−1.22 to 1.90), and cognitive flexibility (−3.83 to 4.23) all span zero and are extremely wide, indicating that the direction of effect, let alone its magnitude, cannot be reliably anticipated in a new study. This uncertainty arises from multiple credible sources: heterogeneity in outcome instruments (e.g., Stroop, Go/No-Go, and Stop Signal tasks are conceptually related but psychometrically distinct measures pooled under ‘inhibitory control reaction time’); variation in ADHD medication status across studies, which may systematically alter baseline executive function and treatment responsiveness; the age range spanning 6–18 years, encompassing substantial developmental differences in executive function maturation; and fundamental differences in the cognitive demands of the exercise modalities themselves, which is precisely the variable under investigation. Subgroup analyses by age and dosage were non-significant, but these tests were severely underpowered with only two studies in several subgroups, and a non-significant result under such conditions should not be interpreted as evidence against moderation. These findings underscore that exercise dose and modality type alone are insufficient to explain between-study variability and that qualitative characteristics of the interventions, including the nature and intensity of cognitive engagement embedded within each exercise type, likely explain a substantial proportion of the observed variance.

### 4.3 Methodological limitations and risk of bias

Several methodological constraints bear on the interpretation of this review’s findings. The near-universal high risk of bias in Domain 2 (deviations from intended interventions) reflects a structural limitation of exercise intervention research: neither participants nor intervention providers can be blinded to group assignment, creating the persistent possibility of performance and expectation bias. This affects all comparisons equally and is a field-wide rather than study-specific problem, but it means that GRADE certainty ratings for all outcomes are capped at moderate at best.

The network meta-analysis was conducted without closed loops in the network geometry for inhibitory control accuracy, which means that formal consistency testing via node-splitting or net heat plots was not possible. Consistency was instead assessed using the SIDE method, which revealed no statistically significant inconsistency (all *p* > 0.05), though the largest numerical discrepancies, particularly between the ACC–MBT and COT–CON comparisons, warrant interpretive caution. The transitivity assumption, while considered plausible based on the clinical and methodological profile of included studies, cannot be formally verified under this network structure.

One included study employed a non-randomised controlled design (Chang et al., 2014). RoB 2 was applied to this study for consistency of reporting; it received the highest overall risk of bias rating in the review. Its influence on pooled estimates for inhibitory control accuracy was examined in sensitivity analyses, which showed stable conclusions, and the study’s inclusion does not materially alter the primary findings. Nonetheless, the inability to apply ROBINS-I, the appropriate tool for non-randomised studies, is acknowledged as a limitation.

The pooling of studies across diverse outcome instruments within each executive function domain introduces additional measurement heterogeneity. Although performance-based measures and behavioural rating scales were not combined, differences between specific neuropsychological tasks (e.g., Stroop versus Stop Signal for inhibitory control) represent a source of construct heterogeneity that statistical approaches cannot fully resolve. Finally, the review is restricted to immediate post-intervention outcomes; the durability of exercise-induced executive function gains, a critical question for clinical application, cannot be addressed from the available data and represents an important direction for future research.

### 4.4 Clinical implications and future research directions

The results indicate that the exercise prescription in children with ADHD should be shifted beyond the generic physical activity recommendation to a more specific choice of physical activity based on the desired cognitive outcomes. Coordinative training activities, like table tennis or rope skipping, seem most promising to families and clinicians focused on improvements in inhibitory control, a core deficit in ADHD related to impulsivity and attention maintenance, according to current evidence. Nevertheless, the high heterogeneity and lack of certainty of evidence suggest that personal responses can differ significantly, which justifies the appeal of Logan and Lim (2025) to consider ADHD subtypes and personal executive function profiles when designing interventions. The lack of significant effects of aerobic exercise by itself, though prominent in current recommendations, indicates that cardiovascular training alone may be inadequate and that the cognitive challenge aspect of exercise may be a key factor in producing executive function benefits. It should also be noted that this review captures only immediate post-intervention effects; whether gains in inhibitory control, particularly those attributed to coordinative training, are sustained beyond the intervention period remains unknown, and clinicians should not assume durability of effect without longitudinal evidence.

Future studies should focus on direct head-to-head comparisons of various exercise modalities in single trials to enhance network connection and minimise the use of indirect comparisons. Whether the apparent superiority of coordinative training in inhibitory control is also relevant to other domains of executive functions and whether integrated strategies involving aerobic training and coordinative training have synergistic effects should be evaluated in large-scale trials. Standardised outcome measurement batteries that measure multiple domains of executive function with both performance-based and rating scale methods would be of significant value to the field and would enable more meaningful cross-study synthesis. Furthermore, mechanistic research that determines whether benefits are mediated by improvements in physical fitness, higher levels of cognitive activity during exercise, or neuroplastic adaptations would maximise intervention design. Lastly, individual difference moderators (such as age, sex, ADHD subtype, baseline executive function severity, and comorbidities) should be investigated to create personalised exercise prescriptions instead of blanket-recommendations.

## 5 Conclusion

This network meta-analysis and systematic review present the most thorough synthesis of comparative evidence about various exercise modalities on executive function in ADHD children to date. Coordinative training proved to be the most effective intervention to enhance the accuracy of inhibitory control, proving stronger than aerobic training, cognitively engaging training, and combined strategies. Combined exercise and mind–body training also demonstrated significant effects in comparison to control conditions, whereas aerobic exercise alone demonstrated non-significant effectiveness. The rigour of such conclusions is, however, dampened by insufficient network connectivity, high levels of between-study heterogeneity, and methodological constraints of exercise-intervention research.

The lack of network meta-analysis of most domains of executive functions because of insufficient studies and weak networks demonstrates a significant gap in evidence base. The field has determined that exercise usually promotes executive functioning in children with ADHD, but does not have comparative evidence to inform choices between various exercise strategies about specific cognitive results. The observed domain-specific effects, where only the accuracy of inhibitory control is robustly demonstrated, undermine assumptions of global executive functioning improvement and highlight the importance of choosing interventions based on specific cognitive outcomes, as opposed to generic exercise guidelines.

To apply to clinical practice, these results indicate that coordinative training tasks that involve dynamic environmental challenge and multifaceted motor-cognitive synthesis ought to be prioritised in case inhibitory control is the focus of intervention. Nonetheless, the variability in treatment response, as represented by broad prediction intervals, underscores the importance of monitoring individual responses and adapting exercise prescriptions accordingly. Large-scale trials directly comparing exercise modalities, standardised outcome measurement methods, systematic exploration of dose–response relationships beyond duration alone, and investigation of individual-difference moderators are needed to permit truly personalised exercise prescription in children with ADHD. Until this evidence is forthcoming, clinicians must consider the modest comparative evidence in support of the use of coordinative training and individual child preferences and capabilities in the recommendation of exercise interventions to aid in the development of executive functions in paediatric ADHD populations.

## Data Availability

The original contributions presented in the study are included in the article/Supplementary material, further inquiries can be directed to the corresponding author.

## Footnotes

1 Accessible at: https://inplasy.com/inplasy-2026-1-0036/.

## References

[ref1] Afonso J. Ramirez-Campillo R. Clemente F. M. Castillo D. García A. García-Romero J. . (2024). The perils of misinterpreting and misusing “publication bias” in meta-analyses: an education review on funnel plot-based methods. Sports Med. 54, 257–269. doi: 10.1007/s40279-023-01927-9 37684502 PMC10933152

[ref2] Al Duhailib Z. Granholm A. Alhazzani W. Oczkowski S. Belley‐Cote E. Møller M. H. (2024). GRADE pearls and pitfalls—part 1: systematic reviews and meta-analyses. Acta Anaesthesiol. Scand. 68, 584–592. doi: 10.1111/aas.14386, 38351600

[ref3] Al-Saad M. S. H. Al-Jabri B. Almarzouki A. F. (2021). A review of working memory training in the management of attention deficit hyperactivity disorder. Front. Behav. Neurosci. 15:686873. doi: 10.3389/fnbeh.2021.686873, 34366803 PMC8334010

[ref4] Amir-Behghadami M. Janati A. (2020). Population, intervention, comparison, outcomes and study (PICOS) design as a framework to formulate eligibility criteria in systematic reviews. Emerg. Med. J. 37:387. doi: 10.1136/emermed-2020-209567, 32253195

[ref5] Augustin M. Mall V. Licata-Dandel M. (2024). ADHD symptoms in middle childhood: the role of child attachment and maternal emotional availability in an inpatient clinical sample. Eur. J. Investig. Health Psychol. Educ. 14, 1572–1584. doi: 10.3390/ejihpe14060104, 38921070 PMC11202776

[ref6] Benzing V. Schmidt M. (2019). The effect of exergaming on executive functions in children with ADHD: a randomized clinical trial. Scand. J. Med. Sci. Sports 29, 1243–1253. doi: 10.1111/sms.13446 31050851

[ref7] Bramer W. M. Rethlefsen M. L. Kleijnen J. Franco O. H. (2017). Optimal database combinations for literature searches in systematic reviews: a prospective exploratory study. Syst. Rev. 6:245. doi: 10.1186/s13643-017-0644-y, 29208034 PMC5718002

[ref8] Brignardello-Petersen R. Mustafa R. A. Siemieniuk R. A. C. Murad M. H. Agoritsas T. Izcovich A. . (2019). GRADE approach to rate the certainty from a network meta-analysis: addressing incoherence. J. Clin. Epidemiol. 108, 77–85. doi: 10.1016/j.jclinepi.2018.11.025, 30529648

[ref9] Brozek J. L. Canelo-Aybar C. Akl E. A. Bowen J. M. Bucher J. Chiu W. A. . (2021). GRADE guidelines 30: the GRADE approach to assessing the certainty of modeled evidence—an overview in the context of health decision-making. J. Clin. Epidemiol. 129, 138–150. doi: 10.1016/j.jclinepi.2020.09.018, 32980429 PMC8514123

[ref10] Büchter R. B. Weise A. Pieper D. (2020). Development, testing and use of data extraction forms in systematic reviews: a review of methodological guidance. BMC Med. Res. Methodol. 20:259. doi: 10.1186/s12874-020-01143-3, 33076832 PMC7574308

[ref11] Bustamante E. E. Davis C. L. Frazier S. L. Rusch D. Fogg L. F. Atkins M. S. . (2016). Randomized controlled trial of exercise for ADHD and disruptive behavior disorders. Med. Sci. Sports Exerc. 48:1397. doi: 10.1249/MSS.0000000000000891, 26829000 PMC4911251

[ref12] Chai W. J. Abd Hamid A. I. Abdullah J. M. (2018). Working memory from the psychological and neurosciences perspectives: a review. Front. Psychol. 9:401. doi: 10.3389/fpsyg.2018.00401, 29636715 PMC5881171

[ref13] Chaimani A. Caldwell D. M. Higgins J. P. T. Salanti G. Dias S. Ades A. E. . (2024). “Chapter 11: undertaking network meta-analyses,” in Cochrane Handbook for Systematic Reviews of Interventions, Version 6.5, eds. Higgins J. Thomas J. Chandler J. . (Chichester, UK: Cochrane).

[ref14] Chang Y.-K. Hung C.-L. Huang C.-J. Hatfield B. D. Hung T.-M. (2014). Effects of an aquatic exercise program on inhibitory control in children with ADHD: a preliminary study. Arch. Clin. Neuropsychol. 29, 217–223. doi: 10.1093/arclin/acu003, 24695590

[ref15] Chang S.-H. Shie J.-J. Yu N.-Y. (2022). Enhancing executive functions and handwriting with a concentrative coordination exercise in children with ADHD: a randomized clinical trial. Percept. Mot. Skills 129, 1014–1035. doi: 10.1177/00315125221098324 35507726

[ref16] Cianchetti C. (2020). Early detection of behavioral and emotional problems in school-aged children and adolescents: the parent questionnaires. Clin. Pract. Epidemiol. Ment. Health 16, 7–16. doi: 10.2174/1745017902016010007, 32508965 PMC7254825

[ref17] Cochrane Training (2023). Using GRADEpro to perform a GRADE… In: YouTube. Available online at: https://www.youtube.com/playlist?list=PLxFw8aTtvq-c90anp9-RN0jD7Gnq_xPxj (Accessed December 20, 2024).

[ref18] Contreras-Osorio F. Campos-Jara C. Martínez-Salazar C. Chirosa-Ríos L. Martínez-García D. (2021). Effects of sport-based interventions on children’s executive function: a systematic review and meta-analysis. Brain Sci. 11:755. doi: 10.3390/brainsci11060755, 34200362 PMC8226694

[ref19] Daly C. H. Neupane B. Beyene J. Thabane L. Straus S. E. Hamid J. S. (2019). Empirical evaluation of SUCRA-based treatment ranks in network meta-analysis: quantifying robustness using Cohen’s kappa. BMJ Open 9:e024625. doi: 10.1136/bmjopen-2018-024625, 31492773 PMC6731799

[ref20] DeMars M. M. Perruso C. (2022). MeSH and text-word search strategies: precision, recall, and their implications for library instruction. J. Med. Libr. Assoc. 110, 23–33. doi: 10.5195/jmla.2022.1283, 35210959 PMC8830400

[ref21] Dettori J. R. Norvell D. C. Chapman J. R. (2022). Fixed-effect vs random-effects models for meta-analysis: 3 points to consider. Glob. Spine J. 12, 1624–1626. doi: 10.1177/21925682221110527, 35723546 PMC9393987

[ref22] Durgut E. Orengul A. C. Algun Z. C. (2020). Comparison of the effects of treadmill and vibration training in children with attention deficit hyperactivity disorder: a randomized controlled trial. NeuroRehabilitation 47, 121–131. doi: 10.3233/NRE-203040, 32741784

[ref23] Freeman S. C. Fisher D. White I. R. Auperin A. Carpenter J. R. (2019). Identifying inconsistency in network meta-analysis: is the net heat plot a reliable method? Stat. Med. 38, 5547–5564. doi: 10.1002/sim.8383, 31647136 PMC6899484

[ref24] Hattabi S. Forte P. Kukic F. Bouden A. Have M. Chtourou H. . (2022). A randomized trial of a swimming-based alternative treatment for children with attention deficit hyperactivity disorder. Int. J. Environ. Res. Public Health 19:16238. doi: 10.3390/ijerph192316238, 36498313 PMC9739874

[ref25] Hernández Ahumada J. Montalva-Valenzuela F. Garrido Chacón S. Jara-Donoso A. Garces Rivas N. Ferrari G. . (2025). Effects of physical activity, exercise and sport on executive function in adults diagnosed with attention deficit hyperactivity disorder: a systematic review. Psychiatry Int. 6:120. doi: 10.3390/psychiatryint6040120

[ref26] Higgins J. P. T. Altman D. G. Gøtzsche P. C. . (2011). The Cochrane collaboration’s tool for assessing risk of bias in randomised trials. BMJ 343:d5928. doi: 10.1136/bmj.d5928 22008217 PMC3196245

[ref27] Higgins J. Thomas J. Chandler J. Cumpston M. Li T. Page M. J. . (2024). Cochrane Handbook for Systematic Reviews of Interventions, Version 6.5. Chichester, UK: Cochrane.

[ref28] Huang C.-Y. Chen W.-F. Tsai C.-L. Chen P.-L. Hsu P.-J. Pan C.-Y. (2025). Effects of inline skating exercise on symptoms, executive functions, and motor proficiency in children with ADHD: a randomized controlled trial. Occup. Ther. Int. 2025:4254970. doi: 10.1155/oti/4254970, 40688800 PMC12271703

[ref29] Huang Z. Li L. Lu Y. Meng J. Wu X. (2024). Effects of rope skipping exercise on working memory and cardiorespiratory fitness in children with attention deficit hyperactivity disorder. Front. Psych. 15:1381403. doi: 10.3389/fpsyt.2024.1381403, 38846914 PMC11153777

[ref30] Hutton B. Salanti G. Caldwell D. M. Chaimani A. Schmid C. H. Cameron C. . (2015). The PRISMA extension statement for reporting of systematic reviews incorporating network meta-analyses of health care interventions: checklist and explanations. Ann. Intern. Med. 162, 777–784. doi: 10.7326/M14-2385, 26030634

[ref31] Jiang Y. Peng H. Song Y. Huang L. Chen H. Li P. . (2025). Evaluating exercise therapies in adolescent idiopathic scoliosis: a systematic review with Bayesian network meta-analysis. PeerJ 13:e19175. doi: 10.7717/peerj.19175, 40183057 PMC11967429

[ref32] Kadri A. Slimani M. Bragazzi N. L. Bragazzi N. Tod D. Azaiez F. (2019). Effect of taekwondo practice on cognitive function in adolescents with attention deficit hyperactivity disorder. Int. J. Environ. Res. Public Health 16:204. doi: 10.3390/ijerph16020204, 30642062 PMC6352161

[ref33] Kofler M. J. Groves N. B. Chan E. S. M. Marsh C. L. Cole A. M. Gaye F. . (2024). Working memory and inhibitory control deficits in children with ADHD: an experimental evaluation of competing model predictions. Front. Psych. 15:1277583. doi: 10.3389/fpsyt.2024.1277583, 38779551 PMC11110569

[ref34] Kostyrka-Allchorne K. Wass S. V. Yusuf H. Rao V. Bertini C. Sonuga‐Barke E. J. S. (2023). Inhibitory deficits and symptoms of attention-deficit hyperactivity disorder: how are they related to effortful control? Br. J. Dev. Psychol. 41, 50–65. doi: 10.1111/bjdp.12432, 36127834 PMC10087402

[ref35] Laloux C. Boulanger B. Bastien P. Carlin B. P. Monseur A. Guillou C. . (2025). Penalized Bayesian methods for product ranking using both positive and negative references. J. Biopharm. Stat. 35, 1126–1142. doi: 10.1080/10543406.2025.2489287, 40492403

[ref36] Leblanc V. Hamroun A. Bentegeac R. Le Guellec B. Lenain R. Chazard E. (2024). Added value of medical subject headings terms in search strategies of systematic reviews: comparative study. J. Med. Internet Res. 26:e53781. doi: 10.2196/53781, 39561364 PMC11615561

[ref37] Lei J. Charman T. Leigh E. Russell A. Mohamed Z. Hollocks M. J. (2022). Examining the relationship between cognitive inflexibility and internalizing and externalizing symptoms in autistic children and adolescents: a systematic review and meta-analysis. Autism Res. 15, 2265–2295. doi: 10.1002/aur.2826, 36196666 PMC10092776

[ref38] Liang X. Qiu H. Wang P. Sit C. H. P. (2022). The impacts of a combined exercise on executive function in children with ADHD: a randomized controlled trial. Scand. J. Med. Sci. Sports 32, 1297–1312. doi: 10.1111/sms.14192, 35611615

[ref39] Liu C. Yang Y. Wong S. H. Wong S. H.-s. Leung A. Sit C. H.-p. (2025). The effects of physical activity on mental health in adolescents with attention-deficit hyperactivity disorder: a randomized controlled trial. Int. J. Behav. Nutr. Phys. Act. 22:47. doi: 10.1186/s12966-025-01745-4, 40247314 PMC12007287

[ref40] Logan N. E. Lim M. (2025). Efficacy of exercise interventions for executive function in children with ADHD: what is the current verdict? Pediatr. Res. 98, 1–2. doi: 10.1038/s41390-025-04646-1 41291221 PMC13102712

[ref41] Ludyga S. Hanke M. Leuenberger R. Bruggisser F. Pühse U. Gerber M. . (2023). Martial arts and cognitive control in children with attention-deficit hyperactivity disorder and children born very preterm: a combined analysis of two randomized controlled trials. Med. Sci. Sports Exerc. 55:777. doi: 10.1249/MSS.0000000000003110, 36728805 PMC10090288

[ref42] Ludyga S. Mücke M. Leuenberger R. Bruggisser F. Pühse U. Gerber M. . (2022). Behavioral and neurocognitive effects of judo training on working memory capacity in children with ADHD: a randomized controlled trial. Neuroimage Clin. 36:103156. doi: 10.1016/j.nicl.2022.103156, 35988343 PMC9402389

[ref43] Mahtani K. R. Heneghan C. Aronson J. (2020). Single screening or double screening for study selection in systematic reviews? BMJ Evid. Based Med. 25, 149–150. doi: 10.1136/bmjebm-2019-111269, 31722997

[ref44] Mathes T. Klaßen P. Pieper D. (2017). Frequency of data extraction errors and methods to increase data extraction quality: a methodological review. BMC Med. Res. Methodol. 17:152. doi: 10.1186/s12874-017-0431-4, 29179685 PMC5704562

[ref45] Mathur M. B. VanderWeele T. J. (2020). Sensitivity analysis for publication bias in meta-analyses. J. R. Stat. Soc. Ser. C. Appl. Stat. 69, 1091–1119. doi: 10.1111/rssc.12440, 33132447 PMC7590147

[ref46] Mbuagbaw L. Rochwerg B. Jaeschke R. Heels-Andsell D. Alhazzani W. Thabane L. . (2017). Approaches to interpreting and choosing the best treatments in network meta-analyses. Syst. Rev. 6:79. doi: 10.1186/s13643-017-0473-z, 28403893 PMC5389085

[ref47] Memarmoghaddam M. Torbati H. Sohrabi M. Ghanbari A. Rezaei M. Shamsi M. . (2016). Effects of a selected exercise programon executive function of children with attention deficit hyperactivity disorder. J. Med. Life 9, 373–379. 27928441 PMC5141397

[ref48] Moseley A. M. Rahman P. Wells G. A. Zadro J. R. Sherrington C. Toupin-April K. . (2019). Agreement between the Cochrane risk of bias tool and physiotherapy evidence database (PEDro) scale: a meta-epidemiological study of randomized controlled trials of physical therapy interventions. PLoS One 14:e0222770. doi: 10.1371/journal.pone.0222770, 31536575 PMC6752782

[ref49] Nair A. S. Borkar N. (2024). Sensitivity and subgroup analysis in a meta-analysis – what we should know? Indian J. Anaesth. 68, 922–924. doi: 10.4103/ija.ija_623_24, 39449843 PMC11498262

[ref50] Nejati V. (2021). Balance-based attentive rehabilitation of attention networks (BARAN) improves executive functions and ameliorates behavioral symptoms in children with ADHD. Complement. Ther. Med. 60:102759. doi: 10.1016/j.ctim.2021.102759 34252575

[ref51] Nejati V. Derakhshan Z. (2021). The effect of physical activity with and without cognitive demand on the improvement of executive functions and behavioral symptoms in children with ADHD. Expert. Rev. Neurother. 21, 607–614. doi: 10.1080/14737175.2021.1912600, 33849353

[ref52] Nie C. Liu X. Provost S. Ren J. (2025). A Markov chain Monte Carlo procedure for efficient Bayesian inference on the phase-type aging model. Stat 8:77. doi: 10.3390/stats8030077

[ref53] Page M. J. McKenzie J. E. Bossuyt P. M. Boutron I. Hoffmann T. C. Mulrow C. D. . (2021). The PRISMA 2020 statement: an updated guideline for reporting systematic reviews. BMJ 372:n71. doi: 10.1136/bmj.n71 33782057 PMC8005924

[ref54] Pan C.-Y. Chu C.-H. Tsai C.-L. Lo S.-Y. Cheng Y.-W. Liu Y.-J. (2016). A racket-sport intervention improves behavioral and cognitive performance in children with attention-deficit/hyperactivity disorder. Res. Dev. Disabil. 57, 1–10. doi: 10.1016/j.ridd.2016.06.009, 27344348

[ref55] Polanin J. R. Pigott T. D. Espelage D. L. Grotpeter J. K. (2019). Best practice guidelines for abstract screening large-evidence systematic reviews and meta-analyses. Res. Synth. Methods 10, 330–342. doi: 10.1002/jrsm.1354

[ref56] Revollo Carrillo N. Gutiérrez-Ruiz K. Iglesias Rodríguez T. Lewis Harb S. (2025). Exploring the potential of Braingame Brian for executive function improvement in Spanish-speaking children with ADHD: a pilot study. Neuropsychol. Rehabil. 35, 1570–1598. doi: 10.1080/09602011.2024.2439614, 39679593

[ref57] Röver C. Knapp G. Friede T. (2015). Hartung-Knapp-Sidik-Jonkman approach and its modification for random-effects meta-analysis with few studies. BMC Med. Res. Methodol. 15:99. doi: 10.1186/s12874-015-0091-1, 26573817 PMC4647507

[ref58] Rožanc I. Mernik M. (2021). “The screening phase in systematic reviews: can we speed up the process?” in Advances in Computers, ed. Hurson A. R. (Cambridge, UK: Elsevier), 115–191.

[ref59] Rücker G. Cates C. J. Schwarzer G. (2017). Methods for including information from multi-arm trials in pairwise meta-analysis. Res. Synth. Methods 8, 392–403. doi: 10.1002/jrsm.1259, 28759708

[ref60] Seide S. E. Röver C. Friede T. (2019). Likelihood-based random-effects meta-analysis with few studies: empirical and simulation studies. BMC Med. Res. Methodol. 19:16. doi: 10.1186/s12874-018-0618-3, 30634920 PMC6330405

[ref61] Smith S. D. Crowley M. J. Ferrey A. Ramsey K. Wexler B. E. Leckman J. F. . (2019). Effects of integrated brain, body, and social (IBBS) intervention on ERP measures of attentional control in children with ADHD. Psychiatry Res. 278, 248–257. doi: 10.1016/j.psychres.2019.06.021, 31233935 PMC6637759

[ref62] Song X. Hou Y. Shi W. Wang Y. Fan F. Hong L. (2025). Exploring the impact of different types of exercise on working memory in children with ADHD: a network meta-analysis. Front. Psychol. 16:1522944. doi: 10.3389/fpsyg.2025.1522944, 39931282 PMC11808027

[ref63] Song F. Khan K. S. Dinnes J. Sutton A. J. (2002). Asymmetric funnel plots and publication bias in meta-analyses of diagnostic accuracy. Int. J. Epidemiol. 31, 88–95. doi: 10.1093/ije/31.1.88, 11914301

[ref64] Sterne J. A. C. Savović J. Page M. J. Elbers R. G. Blencowe N. S. Boutron I. . (2019). RoB 2: a revised tool for assessing risk of bias in randomised trials. BMJ 366:l4898. doi: 10.1136/bmj.l4898 31462531

[ref65] Tao R. Yang Y. Wilson M. Chang J. R. Liu C. Sit C. H. P. (2025). Comparative effectiveness of physical activity interventions on cognitive functions in children and adolescents with neurodevelopmental disorders: a systematic review and network meta-analysis of randomized controlled trials. Int. J. Behav. Nutr. Phys. Act. 22:6. doi: 10.1186/s12966-024-01702-7, 39806448 PMC11731537

[ref66] Tao Y. Zhang Y. Qian H. Cao Z. (2025). Long term effects of physical activity types on executive functions in school aged children. Sci. Rep. 15:30303. doi: 10.1038/s41598-025-09674-9, 40830545 PMC12365328

[ref67] Tseng Y. Chao H. Hung C. (2022). Effect of a strategic physical activity program on cognitive flexibility among children with internet addiction: a pilot study. Children 9:798. doi: 10.3390/children9060798, 35740735 PMC9221991

[ref68] Tsou A. Y. Treadwell J. R. Erinoff E. Schoelles K. (2020). Machine learning for screening prioritization in systematic reviews: comparative performance of abstrackr and EPPI-reviewer. Syst. Rev. 9:73. doi: 10.1186/s13643-020-01324-7, 32241297 PMC7118839

[ref69] Turner R. M. Jackson D. Wei Y. Thompson S. G. Higgins J. P. T. (2015). Predictive distributions for between-study heterogeneity and simple methods for their application in Bayesian meta-analysis. Stat. Med. 34, 984–998. doi: 10.1002/sim.6381, 25475839 PMC4383649

[ref70] Verburgh L. Königs M. Scherder E. J. A. Oosterlaan J. (2014). Physical exercise and executive functions in preadolescent children, adolescents and young adults: a meta-analysis. Br. J. Sports Med. 48, 973–979. doi: 10.1136/bjsports-2012-091441, 23467962

[ref71] Veroniki A. A. Tsokani S. Zevgiti S. Pagkalidou I. Kontouli K.-M. Ambarcioglu P. . (2021). Do reporting guidelines have an impact? Empirical assessment of changes in reporting before and after the PRISMA extension statement for network meta-analysis. Syst. Rev. 10:246. doi: 10.1186/s13643-021-01780-9, 34507621 PMC8434710

[ref72] von Hippel P. T. (2015). The heterogeneity statistic I2 can be biased in small meta-analyses. BMC Med. Res. Methodol. 15:35. doi: 10.1186/s12874-015-0024-z, 25880989 PMC4410499

[ref73] Waffenschmidt S. Sieben W. Jakubeit T. Knelangen M. Overesch I. Bühn S. . (2023). Increasing the efficiency of study selection for systematic reviews using prioritization tools and a single-screening approach. Syst. Rev. 12:161. doi: 10.1186/s13643-023-02334-x, 37705060 PMC10500815

[ref74] Wang J. Yang Y. Li L. Yang X. Guo X. Yuan X. . (2024). Comparative efficacy of physical activity types on executive functions in children and adolescents: a network meta-analysis of randomized controlled trials. J. Sci. Med. Sport 27, 187–196. doi: 10.1016/j.jsams.2023.11.006, 38042755

[ref75] Weber F. Knapp G. Glass Ä. Rücker G. Schumacher M. Bender R. . (2021). Interval estimation of the overall treatment effect in random-effects meta-analyses: recommendations from a simulation study comparing frequentist, Bayesian, and bootstrap methods. Res. Synth. Methods 12, 291–315. doi: 10.1002/jrsm.1471 33264488

[ref76] Zhao J.-G. (2013). Identifying and measuring heterogeneity across the studies in meta-analysis. J. Hand Surg. 38, 1449–1450. doi: 10.1016/j.jhsa.2013.05.020 23790431

[ref77] Zhao L. Agazzi H. Du Y. Meng H. Maku R. Li K. . (2024). A digital cognitive-physical intervention for attention-deficit/hyperactivity disorder: randomized controlled trial. J. Med. Internet Res. 26:e55569. doi: 10.2196/55569, 38728075 PMC11127175

[ref78] Zhu F. Zhu X. Bi X. Li Y. Zhang L. Wang Y. . (2023). Comparative effectiveness of various physical exercise interventions on executive functions and related symptoms in children and adolescents with attention deficit hyperactivity disorder: a systematic review and network meta-analysis. Front. Public Health 11:1133727. doi: 10.3389/fpubh.2023.1133727 37033046 PMC10080114

