# Supplementary material for: Comparative effects of different exercise modalities on executive function in children with attention-deficit/hyperactivity disorder: a systematic review and network meta-analysis
Source: Front Psychol. 2026 Apr 30;17:1786104. doi: 10.3389/fpsyg.2026.1786104 (PMC13171305; doi:10.3389/fpsyg.2026.1786104)
Supplement: Supplementary file 1 [file Supplementary_file_1.docx]

**APPENDIX**

**Appendix 1: Search terms and strategy for all databases**

| **Database** | **Search Strategy** | **Results** |
| --- | --- | --- |
| Web of Science | (TITLE-ABS-KEY (“attention deficit hyperactivity disorder" OR "attention-deficit/hyperactivity disorder" OR "attention deficit disorder" OR "ADHD" OR "hyperkinetic disorder*" OR "minimal brain dysfunction" OR "hyperactivity disorder*”))  AND (TITLE-ABS-KEY (child* OR adolescent* OR pediatric* OR paediatric* OR youth OR "school age*" OR "school-age*" OR teenage* OR juvenile*)) AND (TITLE-ABS-KEY (“executive function*" OR "cognitive control" OR "attention control" OR "inhibitory control" OR "response inhibition" OR "working memory" OR "cognitive flexibility" OR "mental flexibility" OR "task switching" OR "set shifting" OR "cognitive shifting" OR planning OR "decision making" OR "problem solving" OR "self-control" OR "self-regulation" OR "cognitive regulation”) )AND ( TITLE-ABS-KEY ( exercise* OR "physical activity" OR "physical training" OR "fitness training" OR aerobic* OR "cardiovascular training" OR "endurance training" OR running OR jogging OR cycling OR swimming OR "resistance training" OR "strength training" OR "weight training" OR yoga OR "tai chi" OR "martial arts" OR dance OR dancing OR "coordinative training" OR "motor training" OR "balance training" OR exergam* OR "active video game*" OR "cognitively engaging exercise" OR "mind-body" OR "physical intervention*" OR sport* ) )  Refined By: Languages: English. Click to remove this refine from your search. NOT Document Types: Review Article or Meeting Abstract or Early Access or Retracted Publication or Editorial Material. Timespan: 2010-01-01 to 2025-12-01 (Publication Date) | 70 |
| Scopus | ( TITLE-ABS-KEY ( "attention deficit hyperactivity disorder" OR "attention-deficit/hyperactivity disorder" OR "attention deficit disorder" OR "ADHD" OR "hyperkinetic disorder*" OR "minimal brain dysfunction" OR "hyperactivity disorder*" ) AND TITLE-ABS-KEY ( child* OR adolescent* OR pediatric* OR paediatric* OR youth OR "school age*" OR "school-age*" OR teenage* OR juvenile* ) AND TITLE-ABS-KEY ( "executive function*" OR "cognitive control" OR "attention control" OR "inhibitory control" OR "response inhibition" OR "working memory" OR "cognitive flexibility" OR "mental flexibility" OR "task switching" OR "set shifting" OR "cognitive shifting" OR planning OR "decision making" OR "problem solving" OR "self-control" OR "self-regulation" OR "cognitive regulation" ) AND TITLE-ABS-KEY ( exercise* OR "physical activity" OR "physical training" OR "fitness training" OR aerobic* OR "cardiovascular training" OR "endurance training" OR running OR jogging OR cycling OR swimming OR "resistance training" OR "strength training" OR "weight training" OR yoga OR "tai chi" OR "martial arts" OR dance OR dancing OR "coordinative training" OR "motor training" OR "balance training" OR exergam* OR "active video game*" OR "cognitively engaging exercise" OR "mind-body" OR "physical intervention*" OR sport* ) AND TITLE-ABS-KEY ( randomized OR randomised OR "controlled trial*" OR "clinical trial*" OR "intervention study" OR "intervention trial*" OR rct OR "quasi-experimental" OR "pre-post" OR "before-after" OR "control group*" OR "comparison group*" ) AND NOT TITLE-ABS-KEY ( "meta analysis" OR "meta-analysis" OR "systematic review" OR "review" ) ) AND PUBYEAR > 2009 AND PUBYEAR < 2026 AND ( LIMIT-TO ( DOCTYPE , "ar" ) ) AND ( LIMIT-TO ( PUBSTAGE , "final" ) ) AND ( LIMIT-TO ( LANGUAGE , "English" ) ) | 115 |
| PubMed | (("Attention Deficit Disorder with Hyperactivity"[Mesh] OR "attention deficit hyperactivity disorder"[tiab] OR "attention-deficit/hyperactivity disorder"[tiab] OR "attention deficit disorder"[tiab] OR "ADHD"[tiab] OR "hyperkinetic disorder"[tiab] OR "hyperkinetic disorders"[tiab] OR "minimal brain dysfunction"[tiab] OR "hyperactivity disorder"[tiab] OR "hyperactivity disorders"[tiab]) AND ("Child"[Mesh] OR "Adolescent"[Mesh] OR "child"[tiab] OR "children"[tiab] OR "adolescent"[tiab] OR "adolescents"[tiab] OR "pediatric"[tiab] OR "paediatric"[tiab] OR "youth"[tiab] OR "school age"[tiab] OR "school-age"[tiab] OR "teenage"[tiab] OR "teenagers"[tiab] OR "juvenile"[tiab] OR "juveniles"[tiab]) AND ("Executive Function"[Mesh] OR "Memory, Short-Term"[Mesh] OR "Inhibition, Psychological"[Mesh] OR "Attention"[Mesh] OR "executive function"[tiab] OR "executive functions"[tiab] OR "executive functioning"[tiab] OR "cognitive control"[tiab] OR "attention control"[tiab] OR "inhibitory control"[tiab] OR "response inhibition"[tiab] OR "working memory"[tiab] OR "cognitive flexibility"[tiab] OR "mental flexibility"[tiab] OR "task switching"[tiab] OR "set shifting"[tiab] OR "cognitive shifting"[tiab] OR "planning"[tiab] OR "decision making"[tiab] OR "problem solving"[tiab] OR "self-control"[tiab] OR "self-regulation"[tiab] OR "cognitive regulation"[tiab]) AND ("Exercise"[Mesh] OR "Physical Fitness"[Mesh] OR "Sports"[Mesh] OR "Exercise Therapy"[Mesh] OR "Motor Activity"[Mesh] OR "exercise"[tiab] OR "exercises"[tiab] OR "exercising"[tiab] OR "physical activity"[tiab] OR "physical activities"[tiab] OR "physical training"[tiab] OR "fitness training"[tiab] OR "aerobic"[tiab] OR "aerobics"[tiab] OR "cardiovascular training"[tiab] OR "endurance training"[tiab] OR "running"[tiab] OR "jogging"[tiab] OR "cycling"[tiab] OR "swimming"[tiab] OR "resistance training"[tiab] OR "strength training"[tiab] OR "weight training"[tiab] OR "yoga"[tiab] OR "tai chi"[tiab] OR "martial arts"[tiab] OR "dance"[tiab] OR "dancing"[tiab] OR "coordinative training"[tiab] OR "motor training"[tiab] OR "balance training"[tiab] OR "exergaming"[tiab] OR "exergames"[tiab] OR "active video games"[tiab] OR "cognitively engaging exercise"[tiab] OR "mind-body"[tiab] OR "physical intervention"[tiab] OR "physical interventions"[tiab] OR "sport"[tiab] OR "sports"[tiab]) AND ("Randomized Controlled Trial"[Publication Type] OR "Controlled Clinical Trial"[Publication Type] OR "Clinical Trial"[Publication Type] OR "randomized"[tiab] OR "randomised"[tiab] OR "controlled trial"[tiab] OR "controlled trials"[tiab] OR "clinical trial"[tiab] OR "clinical trials"[tiab] OR "intervention study"[tiab] OR "intervention studies"[tiab] OR "intervention trial"[tiab] OR "intervention trials"[tiab] OR "RCT"[tiab] OR "quasi-experimental"[tiab] OR "pre-post"[tiab] OR "before-after"[tiab] OR "control group"[tiab] OR "control groups"[tiab] OR "comparison group"[tiab] OR "comparison groups"[tiab])) NOT ("Meta-Analysis"[Publication Type] OR "Systematic Review"[Publication Type] OR "Review"[Publication Type] OR "Editorial"[Publication Type] OR "Letter"[Publication Type] OR "Comment"[Publication Type] OR "Case Reports"[Publication Type] OR "meta-analysis"[tiab] OR "meta analysis"[tiab] OR "systematic review"[tiab] OR "systematic reviews"[tiab] OR "literature review"[tiab] OR "literature reviews"[tiab] OR "network meta-analysis"[tiab] OR "network meta analysis"[tiab] OR "umbrella review"[tiab] OR "umbrella reviews"[tiab] OR "scoping review"[tiab] OR "scoping reviews"[tiab] OR "narrative review"[tiab] OR "narrative reviews"[tiab] OR "editorial"[tiab] OR "editorials"[tiab] OR "commentary"[tiab] OR "commentaries"[tiab] OR "letter to editor"[tiab] OR "case report"[tiab] OR "case reports"[tiab] OR "case study"[tiab] OR "case studies"[tiab]) Filters applied: Clinical Trial, Randomized Controlled Trial, English, Humans, Child: birth-18 years, Exclude preprints, MEDLINE, from 2010/1/1 - 2025/12/01 | 57 |
| SPORTDiscus with Full Text | (( TI ( "attention deficit hyperactivity disorder" OR "attention-deficit/hyperactivity disorder" OR "attention deficit disorder" OR "ADHD" OR "hyperkinetic disorder*" OR "minimal brain dysfunction" OR "hyperactivity disorder*" ) OR AB ( "attention deficit hyperactivity disorder" OR "attention-deficit/hyperactivity disorder" OR "attention deficit disorder" OR "ADHD" OR "hyperkinetic disorder*" OR "minimal brain dysfunction" OR "hyperactivity disorder*" ) OR KW ( "attention deficit hyperactivity disorder" OR "attention-deficit/hyperactivity disorder" OR "attention deficit disorder" OR "ADHD" OR "hyperkinetic disorder*" OR "minimal brain dysfunction" OR "hyperactivity disorder*" ) )) AND (( TI ( child* OR adolescent* OR pediatric* OR paediatric* OR youth OR "school age*" OR "school-age*" OR teenage* OR juvenile* ) OR AB ( child* OR adolescent* OR pediatric* OR paediatric* OR youth OR "school age*" OR "school-age*" OR teenage* OR juvenile* ) OR KW ( child* OR adolescent* OR pediatric* OR paediatric* OR youth OR "school age*" OR "school-age*" OR teenage* OR juvenile* ) )) AND (( TI ( "executive function*" OR "cognitive control" OR "attention control" OR "inhibitory control" OR "response inhibition" OR "working memory" OR "cognitive flexibility" OR "mental flexibility" OR "task switching" OR "set shifting" OR "cognitive shifting" OR planning OR "decision making" OR "problem solving" OR "self-control" OR "self-regulation" OR "cognitive regulation" ) OR AB ( "executive function*" OR "cognitive control" OR "attention control" OR "inhibitory control" OR "response inhibition" OR "working memory" OR "cognitive flexibility" OR "mental flexibility" OR "task switching" OR "set shifting" OR "cognitive shifting" OR planning OR "decision making" OR "problem solving" OR "self-control" OR "self-regulation" OR "cognitive regulation" ) OR KW ( "executive function*" OR "cognitive control" OR "attention control" OR "inhibitory control" OR "response inhibition" OR "working memory" OR "cognitive flexibility" OR "mental flexibility" OR "task switching" OR "set shifting" OR "cognitive shifting" OR planning OR "decision making" OR "problem solving" OR "self-control" OR "self-regulation" OR "cognitive regulation" ) )) AND (( TI ( exercise* OR "physical activity" OR "physical training" OR "fitness training" OR aerobic* OR "cardiovascular training" OR "endurance training" OR running OR jogging OR cycling OR swimming OR "resistance training" OR "strength training" OR "weight training" OR yoga OR "tai chi" OR "martial arts" OR dance OR dancing OR "coordinative training" OR "motor training" OR "balance training" OR exergam* OR "active video game*" OR "cognitively engaging exercise" OR "mind-body" OR "physical intervention*" OR sport* ) OR AB ( exercise* OR "physical activity" OR "physical training" OR "fitness training" OR aerobic* OR "cardiovascular training" OR "endurance training" OR running OR jogging OR cycling OR swimming OR "resistance training" OR "strength training" OR "weight training" OR yoga OR "tai chi" OR "martial arts" OR dance OR dancing OR "coordinative training" OR "motor training" OR "balance training" OR exergam* OR "active video game*" OR "cognitively engaging exercise" OR "mind-body" OR "physical intervention*" OR sport* ) OR KW ( exercise* OR "physical activity" OR "physical training" OR "fitness training" OR aerobic* OR "cardiovascular training" OR "endurance training" OR running OR jogging OR cycling OR swimming OR "resistance training" OR "strength training" OR "weight training" OR yoga OR "tai chi" OR "martial arts" OR dance OR dancing OR "coordinative training" OR "motor training" OR "balance training" OR exergam* OR "active video game*" OR "cognitively engaging exercise" OR "mind-body" OR "physical intervention*" OR sport* ) )) AND (( TI ( randomized OR randomised OR "controlled trial*" OR "clinical trial*" OR "intervention study" OR "intervention trial*" OR RCT OR "quasi-experimental" OR "pre-post" OR "before-after" OR "control group*" OR "comparison group*" ) OR AB ( randomized OR randomised OR "controlled trial*" OR "clinical trial*" OR "intervention study" OR "intervention trial*" OR RCT OR "quasi-experimental" OR "pre-post" OR "before-after" OR "control group*" OR "comparison group*" ) OR KW ( randomized OR randomised OR "controlled trial*" OR "clinical trial*" OR "intervention study" OR "intervention trial*" OR RCT OR "quasi-experimental" OR "pre-post" OR "before-after" OR "control group*" OR "comparison group*" ) )) NOT (( TI ( "meta-analysis" OR "meta analysis" OR "systematic review*" OR "literature review*" OR "network meta-analysis" OR "network meta analysis" OR "umbrella review*" OR "scoping review*" OR "narrative review*" OR editorial* OR commentary OR commentaries OR "letter to editor*" OR "case report*" OR "case study" OR "case studies" ) OR AB ( "meta-analysis" OR "meta analysis" OR "systematic review*" OR "literature review*" OR "network meta-analysis" OR "network meta analysis" OR "umbrella review*" OR "scoping review*" OR "narrative review*" OR editorial* OR commentary OR commentaries OR "letter to editor*" OR "case report*" OR "case study" OR "case studies" ) OR SU ( "meta-analysis" OR "meta analysis" OR "systematic review*" OR "literature review*" OR "network meta-analysis" OR "network meta analysis" OR "umbrella review*" OR "scoping review*" OR "narrative review*" OR editorial* OR commentary OR commentaries OR "letter to editor*" OR "case report*" OR "case study" OR "case studies" ) OR PT ( editorial OR commentary OR "case report" OR "case study" OR "systematic review" OR "meta-analysis" ) ))  Filters: Peer review Article, Publication range : 2010-2025, Language: English ; Publication Type: Academic Journal; Document type: Article | 12 |
